# Discovery of novel SND1 inhibitors by in silico–based molecular docking and dynamics simulation methods for managing hepatocellular carcinoma

**DOI:** 10.1038/s41598-025-14878-0

**Published:** 2025-08-21

**Authors:** Yuvaraj Ravikumar, Sirajunnisa Abdul Razack, Shilpa Sivashankar, Sirichai Srichairatanakool, Pimpisid Koonyosying, Somdet Srichairatanakool

**Affiliations:** 1 Department of Biotechnology, Acharya Institute of Technology, Soladevanahalli, 560107 Karnataka India; 2 Division of Hematology, Department of Internal Medicine, Chiang Mai University, Chiang Mai, 50200 Thailand; 3 College of Public Health Sciences, Chulalongkorn University, Bangkok, 10330 Thailand; 4 Department of Biochemistry, Faculty of Medicine, Chiang Mai University, Chiang Mai, 50200 Thailand

**Keywords:** Hepatocellular carcinoma, SND1 inhibitor, In silico virtual screening, Molecular dynamics, Simulation, Pharmacokinetic properties, Biochemistry, Computational biology and bioinformatics

## Abstract

Hepatocellular carcinoma (HCC) is one of the life–threatening cancers in the current decade. Earlier reports suggest that protein arginine methyl transferase 5 (PRMT5) is also linked with HCC, and the symmetrically dimethylated arginine marks created by PRMT5 are read by the Staphylococcal nuclease domain-containing protein 1 (SND1), which eventually can drive HCC development. Indeed, as no inhibitors have been ascertained for SND1, we aimed to screen for new inhibitors with specificity and high affinity towards the active site of SND1. In this study, we first used the Gene Set Cancer Analysis (GSCA) dataset to evaluate the expression of SND1 in HCC. Accordingly, SND1 is the highly overexpressed gene in HCC and its overexpression affects the overall survival of HCC patients. Then, we employed a virtual screening approach to identify potential inhibitors from the ASINEX database. We utilized molecular docking to single out the potential top hits based on the docking score and mode of interaction with the SND1 active site. In the prediction, we found [4–(5,6,7,8–tetrahydro–4 H–cyclohepta[c][1,2]oxazol–3–yl)piperidin–1–yl]–[4–(trifluoromethyl)phenyl]methanone (TOP1: -10.4 kcal/mol) and 1–[2–hydroxy–2–(1–methylsulfonyl–3,4–dihydro–2 H–quinolin–6–yl)ethyl]–4–(4–methylphenyl)piperidin–4–ol (TOP2: -10.3 kcal/mol) as promising candidates than reference compound (STD: -4.6 kcal/mol). Further molecular dynamics simulations and a pharmacokinetic properties study indicate that TOP2 exhibits a more stable binding with the SND1 active site and complies with Lipinski’s rule of five for drug-likeness, with no observed toxicity and good pharmacokinetic properties.

**Supplementary Information:**

The online version contains supplementary material available at 10.1038/s41598-025-14878-0.

## Introduction

In the current decade, liver cancer is the world’s third leading cause of cancer death, and hepatocellular carcinoma (HCC) is considered to be the most common form of liver cancer, accounting for 75–90% of liver cancers^1^. Factors such as alcohol abuse, obesity, hepatitis infection, type 2 diabetes, and carcinogen exposure are all considered high-risk factors for developing HCC^2,3^. The continuous aggregation of epigenetic and genetic abnormalities due to the risk factors leads to tumor suppressor genes’ inactivation or activation of the oncogenes causing HCC^4–6^. Due to the late-stage diagnosis and restricted understanding of the molecular drivers of HCC, the mortality rate tends to remain often high, with almost no cure for survival. The only hope is liver transplantation, and fortunately, the number of liver transplants has risen to ~ 18% in the past five years in the U.S. In the last year alone, more than 9400 transplantations have been reported^7^. Nevertheless, the difference in the number of donors to transplantation ratio limits the transplantation process. In-depth knowledge of epigenetic and genetic drivers of HCC must be well-explored, particularly about enzymes/proteins involved in posttranslational modifications (PTM). The three classes of regulatory proteins: writers, readers, and erasers contribute to epigenetic regulation, where writers are enzymes that perform methylation and acetylation in DNA and histones, while readers involve themselves in identifying such chemical modifications, and erasers help to control or modulate the gene expression^8,9^. Since epigenetic modifications are reversible and provide an opportunity for therapeutic development, various drugs that target epigenetic regulations have been developed and have proven successful in cancer treatment^10,11^. For instance, 5-azacytidine (5-aza), one of the FDA–approved epigenetic drugs, crosslinks with DNA methyltransferase 1(DNMT1) and guides it for degradation. Similarly, Tazemetostat, another FDA–approved EZH2 drug, has been used for treating metastatic epithelioid sarcoma^12^.

In humans, arginine methylation, being a ubiquitous PTM, exists in three forms: asymmetric dimethyl arginine (ADMA), symmetric dimethylarginine (SDMA), and monomethyl arginine (MMA)^5,13^. Among these unique methyl marks, SDMA marks are generated most by protein arginine methyl transferase 5 (PRMT5)^14,15^. Importantly, recent studies show that PRMT5 is overexpressed in HCC and strongly correlates with prognosis. Elevated PRMT5 levels in the form of mRNA/protein correlate with higher recurrence and lower survival rates^16–18^. Furthermore, it is still elusive whether the PRMT5 activity under hepatic conditions is oncogenic, and its role in promoting HCC has to be envisaged. SDMA marks generated by PRMT5 bind with specific proteins called effector proteins. These effector proteins are primarily involved in relaying signal information within cells, and specific regions called the “Tudor domain” are responsible for reading the SDMA marks^19^. Staphylococcal nuclease domain-containing protein 1 (SND1) is one such effector protein consisting of four tandem Staphylococcal nuclease (SN)–like domains and a C-terminal Tudor domain, which is present before the fifth SN domain. SND1, abundantly found in the pancreas and liver tissues, binds to the SDMA marks through an aromatic cage α-helix linker. Recently, the mechanism of how SND1 becomes oncogenic and acts as a molecular drive, particularly in HCC, has been identified. A genetically engineered mouse model study confirmed the direct evidence of SND1 overexpression in the liver, leading to spontaneous carcinoma and aggravated tumorigenic response^20^. More importantly, the studies found that the Tudor domain of the SND1 is responsible for reading the marks generated by PRMT5 and contributes to the SND1 becoming oncogenic. In addition, the analysis of immuno–stained tissue microarray samples revealed the overexpression of SND1 in 74% of HCC patients^21,22^. These findings are encouraging and spurred the researchers to develop small-molecule inhibitors that could target the Tudor domain and α-helix linker, which act as an active site in SND1 protein as a novel treatment of HCC.

**Fig. 1:**
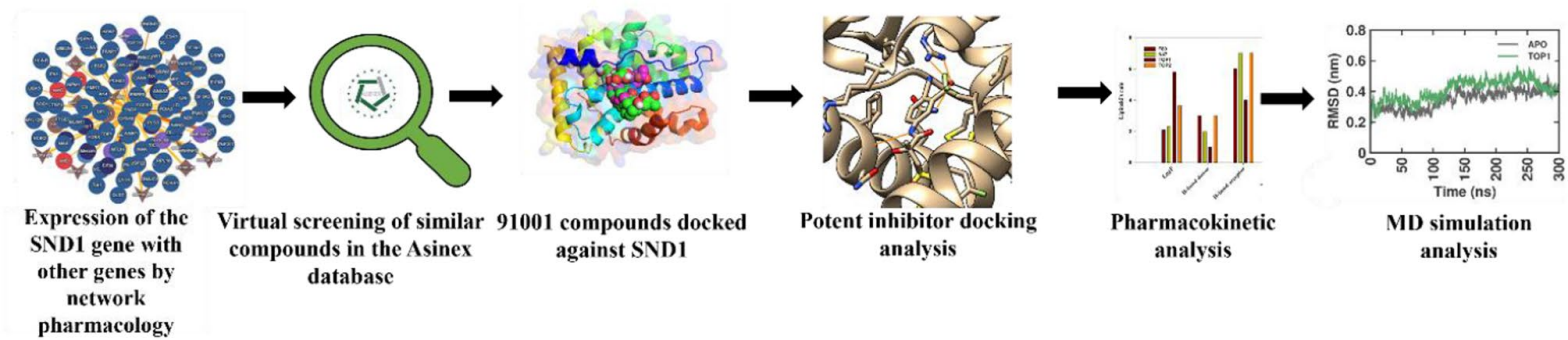
Graphical abstract and summary of the proposed work demonstrating the discovery of novel potential SND1 inhibitors based on molecular docking and dynamic simulation studies. Abbreviation: SND1 = Staphylococcal nuclease domain-containing protein 1.

SND1 can be notably crucial for drug discovery as they have a specific docking site for binding to the SDMA marks left by PRMT5. Therefore, in this study, we intended to target the active site of SND1 and utilized the ASINEX database, where a library of 91,001 compounds was screened to identify a novel group of SND1 inhibitors. The top 15 docked complexes were filtered based on the docking score. The top 2 best docking score compounds and the standard SDMA are subjected to investigation of pharmacokinetic and toxicity properties. Further, MMPBSA energy calculations and MD simulations were also performed to determine the potential SND1 inhibitors. The results obtained from calculated binding energies and MD simulations were compared with the SDMA (hereafter referred to as STD), a natural substrate for the SND1. The overall work plan is depicted in Fig. 1. For HCC management, the results obtained from this study are the first to report the identification of small molecule SND1 inhibitors using in silico computational methods, which would be highly useful for mitigating HCC.

## Results

### Significance of SND1 gene expression in cancer

**Fig. 2:**
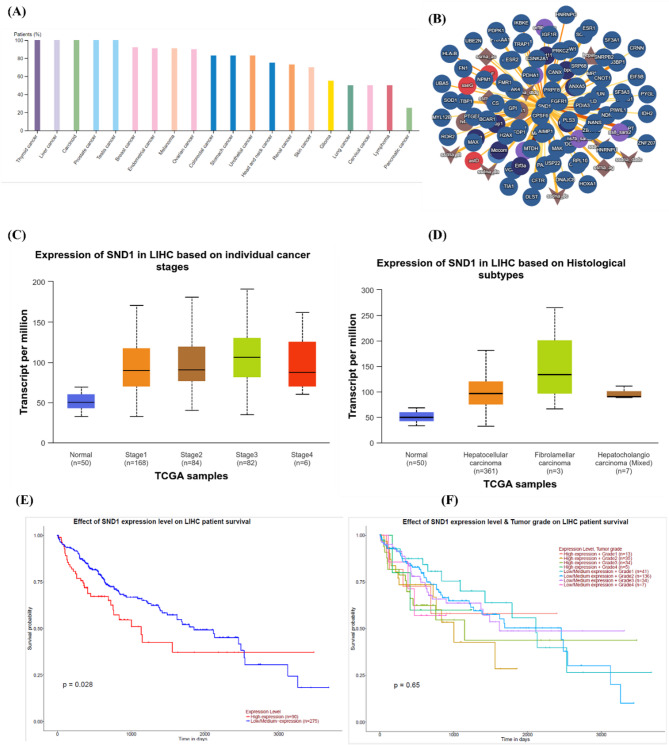
Protein-protein interaction and expression of SND1 in HCC. (**A**) Percentage of SND1 expression in various types of cancers. (**B**) SND1 interacts with different proteins that are involved in numerous cancer types. (**C**) Bar graph indicating the overexpression profile of SND1 in liver hepatocellular carcinoma (LIHC) during different cancer stages (**D**), bar graph indicating the overexpression profile of SND1 in histological subtypes. (**E**) Line graph showing the relationship between SND1 overexpression and patients’ overall survival. (**F**) Line graph showing the relationship between SND1 overexpression during different tumor grades and patients’ overall survival. Abbreviation: HCC = hepatocellular carcinoma; LIHC = liver hepatocellular carcinoma; SND1 = Staphylococcal nuclease domain-containing protein 1.

We used Gene Set Cancer Analysis (GSCA) datasets to unravel the relationship between SND’s gene expression, its interactions with other proteins, and mutation effects in HCC. The GSCA dataset revealed that SND1 is one of the eminently mutated genes in HCC, bearing 1.5% somatic mutation frequency, and SND1 overexpression is predominantly observed in liver cancer (Fig. 2A). Next, the interaction of SND1 with other proteins was identified. We found that SND1 could interact with more than 50 proteins responsible for driving various types of cancers (Fig. 2B). Further, the expression level of SND1 in HCC–affected patients at multiple stages and histological subtypes was analyzed. As shown in Fig. 2C and D, we found that around 1–fold higher expression was observed during stage-III liver cancer. Elevated expression was noticed in various tissue types of HCC, predominantly in fibrolamellar tissues. This observation is crucial as determining the overexpression of SND1 in the earlier stages could be a potential biomarker for HCC development. Further, the liaison between overall survival and SND1 expression in HCC and across different tumor grades was evaluated. The GSCA dataset analysis showed that higher expression of SND1 significantly reduced the overall survival rate in HCC patients. A 50% reduction in survival months was observed (Fig. 2E). More interestingly, the SND1 gene overexpression drastically shortened the survival months in grade III tumors of liver cancer patients (Fig. 2F).

### Virtual screening and molecular docking analysis

**Fig. 3:**
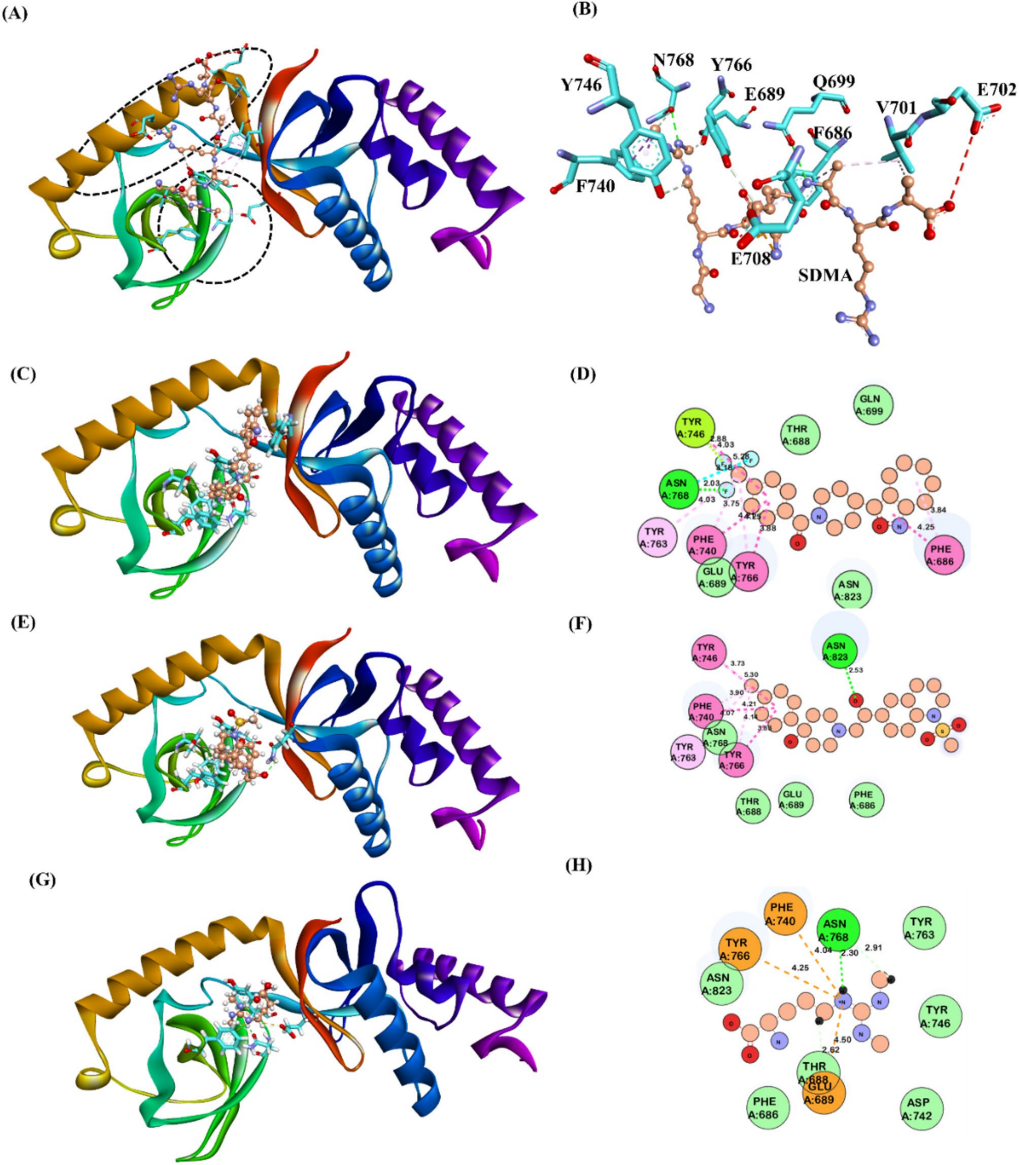
Archetypical cartoon representation of the crystal structure 3OMC. (**A**) Cartoon structure representing the SND1 with SDMA containing PIWIL peptide. (**B**) 3D representation depicting the ligand (SDMA) bound with the SND1 active site. (**C**) 3D–cartoon representation of 3OMC docked with the TOP1. (**D**) 2D–cartoon representation of 3OMC docked with the TOP1. (**E**) 3D–cartoon representation of 3OMC docked with the TOP2. (**D**) 2D–cartoon representation of 3OMC docked with the TOP1. (**F**) 3D–cartoon representation of 3OMC docked with the STD. (**D**) 2D–cartoon representation of 3OMC docked with the STD. (For interpretation of the ligand interaction with the active site, the Cyan stick represents the active site residues interacting, and the orange-scaled ball and disc (2D–right side) represent each respective ligand. Abbreviation: SND1 = Staphylococcal nuclease domain–containing protein 1; TOP1 = [4–(5,6,7,8–tetrahydro–4 H–cyclohepta[c][1,2]oxazol–3–yl)piperidin–1–yl]–[4–(trifluoromethyl)phenyl]methanone; TOP2 = 1–[2–hydroxy–2–(1–methylsulfonyl–3,4–dihydro–2 H–quinolin–6–yl)ethyl]–4–(4–methylphenyl)piperidin–4–ol. Figures were generated by using BIOVIA DISCOVERY STUDIO.

The protein-protein interaction and gene expression analysis using GSCA reveals that SND1 is an assuring target in HCC to improve patients’ overall survival^21^. The Tudor domain and α–helix present in SND1 are primarily accountable for reading the STD marks produced by PRMT5, and the further progression of molecular events could drive liver cancer. Therefore, small-molecule inhibitors’ inhibition of the cited active site will disrupt the ability to read STD during the PRMT5–SND1 interaction. Thus, the crystal structure of SND1, Protein Data Bank (PDB) ID: 3OMC, was employed for docking studies. Since the SND1 active site has high specificity with STD binding, STD was chosen as the reference ligand for this study. Notably, no small molecule inhibitors that target and bind with the SND1 active site are available; hence, only one compound (STD) was taken as a reference. We harnessed the ASINEX database compounds ELITE and SYNERGY library to identify and develop a potential specific inhibitor to combat HCC. 91,001 compounds were retrieved and docked in the SND1 active site region. After docking, the compounds that exhibited the best docking scores, along with STD, were selected and ranked according to the docking score. The TOP 15 compounds, considered potential hits, were taken, and their binding interactions with the active sites were analyzed further. Proper binding poses with the active site were detected in all the TOP 15 compounds. Table 1 displays the top 15 compounds’ docking scores and interaction type with active site residues. The docking scores were calculated using AutoDock Vina, and the docking score range was between − 10.4 and − 9.5 kcal/mol. The molecular interactions that encompass both covalent and noncovalent interactions involved in the binding of TOP 15 compounds are summarized in Table 1. In TOP3, TOP5, and TOP15, we found that H-bond mediated interactions were missing, and only various hydrophobic interactions were formed between each ligand and the active site residues. More importantly, compounds [4–(5,6,7,8–tetrahydro–4 H–cyclohepta[c][1,2]oxazol–3–yl)piperidin–1–yl]–[4–(trifluoromethyl)phenyl]methanone (TOP1) and 1–[2–hydroxy–2–(1–methylsulfonyl–3,4–dihydro–2 H–quinolin–6–yl)ethyl]–4–(4–methylphenyl)piperidin–4–ol (TOP2) exhibited best docking scores of -10.4 kcal/mol and − 10.3 kcal/mol when compared to the STD (-4.6 kcal/mol). Both TOP1 and TOP2 bind to the residues that bind with STD. This is validated with earlier published reports where the authors confirmed that Phe686, Glu689, Gln699, Val701, Glu708, Phe740, Tyr746, Tyr766, and Asn768 from the canonical Tudor domain and α-helix region. Residues Thr688 and Asn823 from the SN domain of the SND1 protein play a crucial role in binding with the SDMA part of PIWIL1. Accordingly, from the docking results, we observed that Asn768 forms an H-bond with TOP1 and Asn823 forms an H–bond interaction with TOP2. Similarly, Asn768 forms an H-bond association with our reference docked compound STD. It is also inevitable to highlight that the fluorine atom present in TOP1 forms a more vital halogen-bond interaction with Asn768 (Fig. 3). In addition, the hydrophobic, π cation–π, and π–π interactions formed between STD and compounds from TOP 1 to TOP 15 are depicted as 3D representation in Figure S1 and summarized in Table 1. The 2D structural representation of STD, TOP1 and TOP2 is depicted in Figure S2. Furthermore, albeit TOP15 compounds have some structural similarities, the slight variations in their functional groups lead to differences in predicted docking scores.

**Table 1:** A summary of the top Docking score compounds docked with SND1 and their interactions with the active site.

Ligand name	PubChem ID	Number of H bond(s)	Interacting residues	Number of non-covalent interactions	Interacting residues	Vina score (kcal/mol)
STD	169,148	1	N768	1	Y766	-4.6
TOP1	92,413,470	1	N768	9	F686, Y746, F740, Y766	-10.4
TOP2	46,954,798	1	N823	8	F740, Y746, Y763, Y766	-10.3
TOP3	110,212,857	0	NA	9	F740, Y746, Y763, Y766, N823	-10.2
TOP4	92,558,433	1	N823	7	F740, Y746, Y763, Y766	-10.2
TOP5	16,671,632	2	Q699, Y766	13	F686, F740, Y746, Y763, Y766, N823	-10.1
TOP6	16,669,238	2	Y746, Y766	7	F686, Y766, F740, Y746	-9.9
TOP7	95,803,799	0	0	10	F686, L707, E708, Y746, Y763, Y766, F740	-9.9
TOP8	110,220,686	2	Y746, Y766, N768	6	F686, F740, Y746, Y766	-9.8
TOP9	46,954,920	1	Q699, N768	5	F686, Y746, Y766, F740	-9.8
TOP10	92,558,071	2	Q689, N823	12	F686, F740, Y746, Y 763, Y766	-9.7
TOP11	92,418,724	1	Y766	10	F686, F740, Y746, Y763, Y766	-9.6
TOP12	110,218,978	2	Y766, N823	6	F686, Y746, Y766, F740	-9.6
TOP13	92,419,523	0	NA	7	F686, Y766, F740, Y746, Y66	-9.6
TOP14	92,555,944	3	Q689, Y766, N768	9	F686, F740, Y746, Y763, Y766	-9.6
TOP15	124,350,199	2	Q689, Y766	11	F686, Y746, Y763, Y766, F740	-9.5

SND1 = Staphylococcal nuclease domain–containing protein 1; TOP1 = [4–(5,6,7,8–tetrahydro–4 H–cyclohepta[c][1,2]oxazol–3–yl)piperidin–1–yl]–[4–(trifluoromethyl)phenyl]methanone; TOP2 = 1–[2–hydroxy–2–(1–methylsulfonyl–3,4–dihydro–2 H–quinolin–6–yl)ethyl]–4–(4–methylphenyl)piperidin–4–ol.

### Physiochemical characterization and pharmacokinetic analysis

The prediction of physiochemical properties of putative potential hits earned from docking studies is crucial for assessing their drug–likeness and enabling them to be used for clinical trials. Therefore, the TOP1 and TOP2 compounds are analyzed for drug likeness. Based on the ‘Lipinski Rule of Five,’ the SwissADME prediction revealed that the logP and molecular weight for TOP1 and TOP2 are less than five and under 500 Da. The number of rotatable bonds found in TOP1 was 4, and in TOP2 was 5. Another important drug-likeness property that determines the absorption efficiency of the drug molecule is the total polar surface area (TPSA), and the values were lower than 140 for TOP1 and TOP2 compounds. Next, in the adsorption criteria, the gastrointestinal (GI) absorption was predicted to be high, along with the P-glycoprotein (P–gp) uptake, which shows that these compounds possess good absorbability in the intestine and could well uptake by the P–gp receptors. The cytochrome P-450 (CYP)-family enzymes that play a vital role in TOP1 and TOP2 metabolism are investigated, and their effects are presented in Table S1. TOP2 exhibits itself as a non–inhibitor for most of the CYP450 inhibitors when compared to TOP1. Total clearance (TC), related to a compound’s bioavailability and half-life, is imperative for deciding the dose concentration and dose interval of a chosen drug. Concerning clinical significance, the TC values of TOP1 and TOP2 were 3.090 and 10.213, respectively. Based on the TC values, it is evident that TOP2 has better excretion ability than TOP1. Besides pharmacokinetics, to circumvent the final step defeat during drug development, the prediction of compound toxicity is of utmost importance. Hence, the toxicity endpoints for TOP1 and TOP2 were predicted using the Pro–Tox II webserver. The toxicity profile includes various organ toxicity and adverse effects on the nuclear receptor signalling pathway and stress response pathway, as described in. The results showed that the overall predicted toxicity for TOP1 comes under class 3, while for TOP2, it falls into class 5. The estimated median lethal dose (LD_50_) for TOP1 is 180 mg/kg, while for TOP2, the value is much higher, 2600 mg/kg. Neither compound exhibited hepatotoxicity, immunotoxicity, mutagenicity, and cytotoxicity. Furthermore, both compounds do not show adverse effects on reproductive and stress-related pathway proteins. The pan assay interference compounds (PAINS) analysis for TOP1 and TOP2 also reveals the tested compounds have succeeded in that parameter, enabling them to be a good choice for the further drug development process. Furthermore, the biological activities of TOP1 and TOP2 were predicted using an online web server (PASS). This evaluation is crucial as it helps identify the outcome of key biological activities in the human body if taken as a drug. As summarised in Table S2, the prediction shows that our putative drug compounds (TOP1 and TOP2) possess antiviral activity against hepatitis C virus (HCV), human immunodeficiency virus (HIV), Rhinovirus, and Middle East Respiratory Syndrome (MERS). Notably, TOP2 shows inhibitory activity against Beta coronavirus England 1, Human coronavirus EMC, where the (aPa) active probability is higher than the (bPi) inactive probability. In addition, both compounds can counteract neurodegenerative diseases, as they exert antidepressants, analgesics, and antipsychotics in common. The other common bioactivities that are predicted using the PASS server are listed in Table S2.

### Molecular dynamics simulations

#### Stability analysis

**Fig. 4:**
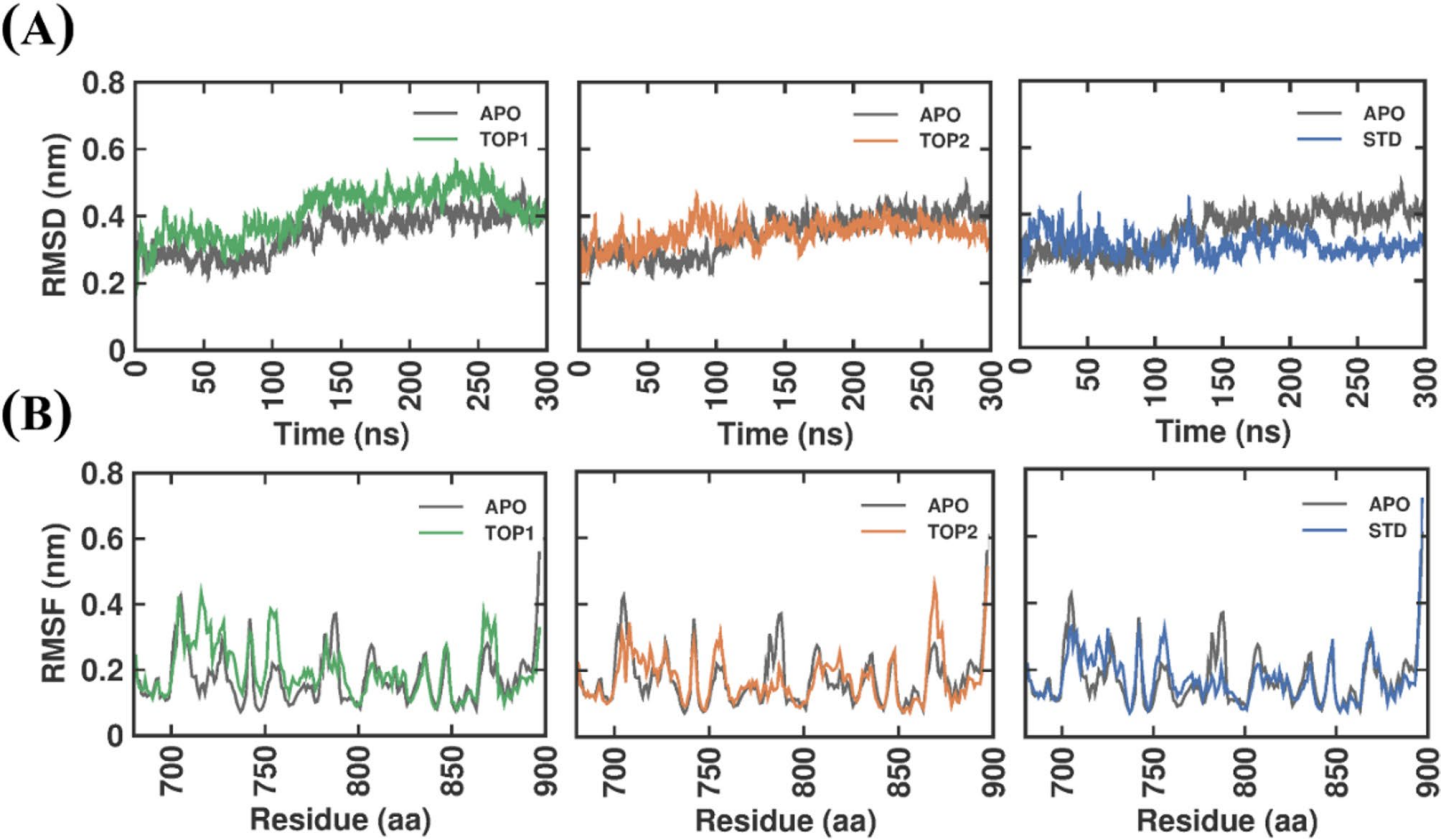
The RMSD and RMSF plot of docked complexes generated through MDS at 300 ns. (**A**) The above plot depicts the RMSD of the SND1 (APO) complex with TOP1, TOP2, and STD. (B) The plot below depicts the RMSF of SND1 (APO) and the complex with TOP1, TOP2, and STD. Abbreviation: RMSD = root–mean–square deviation; RMSF = root–mean–square fluctuation; SND1 = Staphylococcal nuclease domain–containing protein 1; TOP1 = [4-(5,6,7,8–tetrahydro–4 H–cyclohepta[c][1,2]oxazol–3–yl)piperidin–1–yl]–[4–(trifluoromethyl)phenyl]methanone; TOP2 = 1–[2–hydroxy–2-(1–methylsulfonyl–3,4–dihydro–2 H–quinolin–6–yl)ethyl]–4-(4–methylphenyl)piperidin-4–ol. Figures were generated by using QTGRACE Version 5.1.22–.

The Molecular dynamics simulations (MDS) were performed for 300 ns, allowing the system to attain an equilibrium state. The trajectory data obtained from the equilibrated state were taken for comprehensive analysis of four systems: (i) SND1–APO, (ii) SND1 complexed with STD, (iii) SND1 complexed with TOP1, and (iv) SND1 complexed with TOP2. The root-mean-square deviation (RMSD) and root–mean–square fluctuation (RMSF) analyses were performed to assess the stability of the four systems. The RMSD assesses the strength of the protein–ligand complex by investigation of any substantial dynamic changes during the equilibration period. Figure 4A shows the RMSD values of APO, TOP1, TOP2, and STD throughout 300 ns. Although the RMSD of TOP1 and TOP2 fluctuated slightly initially, the SND1-TOP2 complex attained a steady-state equilibrium after 100 ns. In comparison, mild fluctuations (~ 0.3 to ~ 0.5 nm) were seen in the SND1–TOP1 system during a 100–150 ns. In the SND1–STD complex, from 0 to 100 ns, no distinct differences in RMSD were observed, and minimal fluctuations of around ~ 0.15 nm were noted from 100 to 300 ns. SND1–APO, SND1–TOP1, SND1–TOP2 and SND1–STD were found to have average RMSD values of 0.35 ± 0.06 nm, 0.41 ± 0.07 nm, 0.35 ± 0.04 nm and 0.31 ± 0.04 nm, respectively. In all the ligand-bound complexes, the RMSD deviation values do not exceed 0.5 nm, showing they are stable in the simulated trajectory. Next, to discern the relationship between each complex’s residue flexibility and stability, the RMSF value of individual residues, which denotes the deviation of its position from the reference position over the simulation time, was determined and presented in Fig. 4B. When compared to the SND1–APO form, the residue position at 756 in SND1–TOP1, SND1–TOP2, and SND1–STD showed higher fluctuations where ~ 0.17 and ~ 0.12 nm increased RMSF values were observed in TOP1 and STD-bound complex. TOP2 showed lower fluctuations (~ 0.10 nm) when compared to TOP1. Additionally, much higher fluctuations of around ~ 0.25 nm were in the regions corresponding to residues between 715 and 718 in the SND1–TOP1 system. Nevertheless, in both TOP1 and TOP2–bound complexes, the RMSF deviations do not exceed > 3.0 nm, which indicates that the ligand–bound complex remains stable during the MD simulation. It is equally important to note that the RMSF values for catalytically important residues such as 768, 688, 740, 742, 746, 763, 766, and 823 do not show any substantial fluctuation, as the deviation was much lower than 0.1 nm. The average RMSF values for SND1–APO, SND1–TOP1, and SND1–TOP2 were 0.17 ± 0.08 nm, 0.20 ± 0.08 nm, 0.17 ± 0.07 nm and 0.17 ± 0.08 nm, respectively. The findings show that the overall RMSF distribution was not substantially changed by the SND1–APO, SND1–TOP1, SND1–TOP2, and SND1–STD complex.

#### Structure compactness analysis

**Fig. 5:**
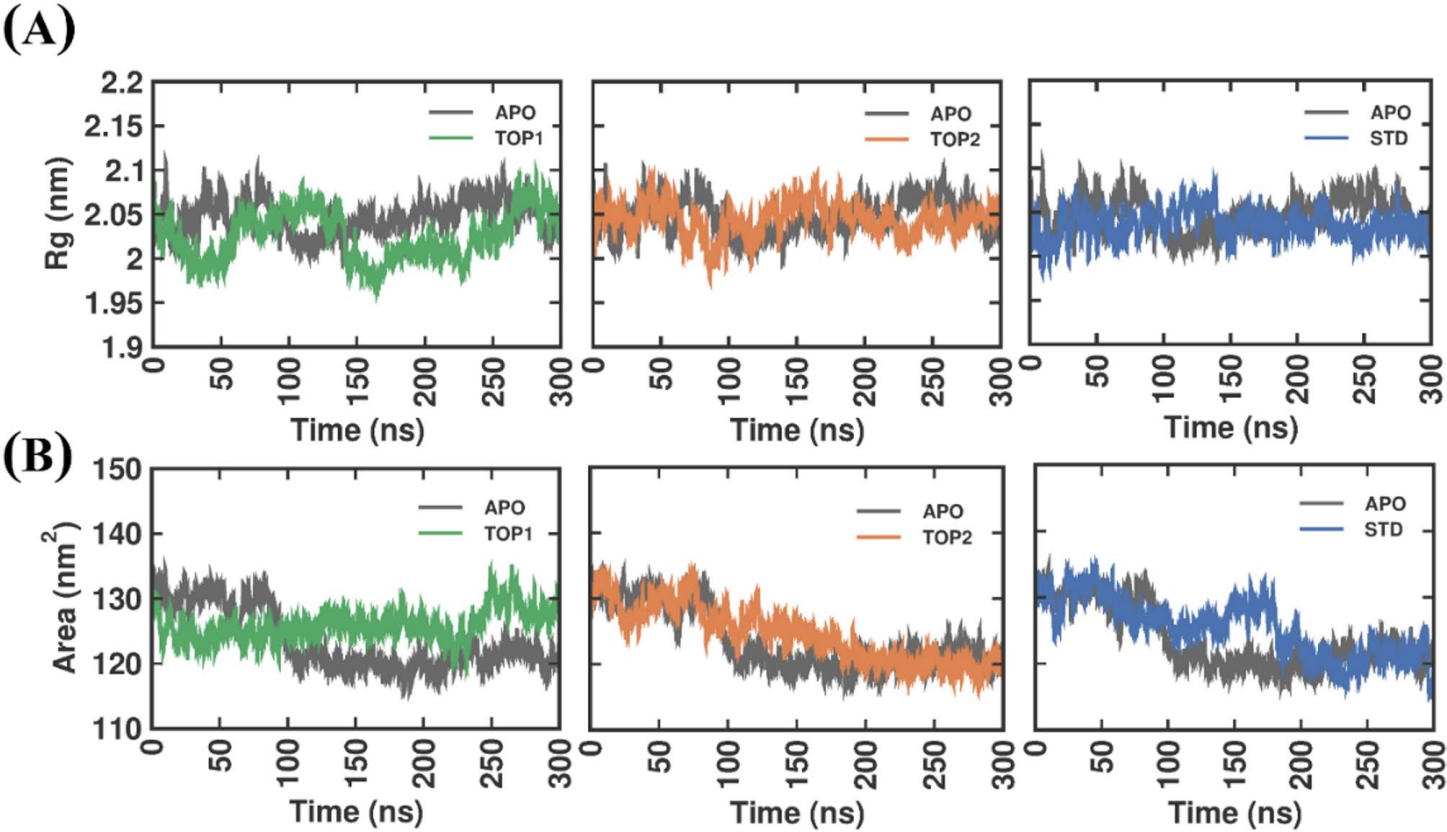
Rg and SASA plot of docked complexes generated through MDS at 300 ns. (**A**) The above plot depicts the Rg of SND1 (APO), which is complex with TOP1, TOP2, and STD. (**B**) The plot below depicts the solvent accessible surface area (SASA) of SND1 (APO), which is also complex with TOP1, TOP2, and STD. Abbreviation: MDS = molecular dynamics simulations; RMSD = root–mean–square deviation; Rg = Radius of Gyration; SASA = solvent accessible surface area; SND1 = Staphylococcal nuclease domain–containing protein 1; TOP1 = [4–(5,6,7,8–tetrahydro–4 H–cyclohepta[c][1,2]oxazol–3–yl)piperidin–1–yl]–[4–(trifluoromethyl)phenyl]methanone; TOP2 = 1-[2-hydroxy-2-(1-methylsulfonyl-3,4–dihydro–2 H–quinolin–6–yl)ethyl]–4–(4–methylphenyl)piperidin–4–ol. Figures were generated by using QTGRACE Version 5.1.22.

The Radius of Gyration (Rg) analysis, as shown in Fig. 5A, demonstrates the fluctuations for all four systems described above. All the fluctuations were compared with the APO form. In SND1–TOP1, at 0 ns, the fluctuations approximately start at ~ 2.05 nm and end at around ~ 2.05 nm at 300 ns. Roughly 0.05 nm differences were observed between the simulation’s trajectory, where at 50, 155, and 240 ns, the Rg value was reduced for 0.07 nm. In contrast, decreasing fluctuations were observed in the SND1–TOP2 complex, where the Rg values tend to be more similar to the APO form throughout the 300 ns period. Similar observations were seen in the SND1–STD complex as well. The average Rg values for SND1–APO, SND1–TOP1, SND1–TOP2, and SND1–STD complex were 2.05 ± 0.02 nm, 2.03 ± 0.03 nm, 2.04 ± 0.02 nm, and 2.04 ± 0.02 nm, respectively. As illustrated, all complex systems showed nearly identical values, suggesting that the protein–bound with each ligand is compact and intact. The solvent accessible surface area (SASA) plot (Fig. 5B) initially illustrates lower values for the SND1–TOP1 system, where the values reduced from ~ 130 to ~ 120 nm from 0 to 200 ns. Increased values were seen between 200 and 250 ns, reaching ~ 130 nm and remaining stable afterward. In contrast, SND1–TOP2 SASA values, after showing initial stability until 75 ns, the values got lowered linearly from ~ 130 nm to ~ 120 nm from 75 to 300 ns. A similar reduction pattern was observed in the SND1-STD complex, although a minimal rise and fall in the SASA value were observed from 150 to 180 ns. Despite these slight deviations, the average values for SND1–APO, SND1–TOP1, SND1–TOP2, and SND1–STD complex were 123.54 ± 5.01 nm, 125.93 ± 2.9 nm, 124.48 ± 4.5 nm, and 125.40 ± 4.5 nm, respectively, which indicates the complexes exhibited fair equilibration with no discernible oscillations.

#### Hydrogen bond analysis

**Fig. 6:**
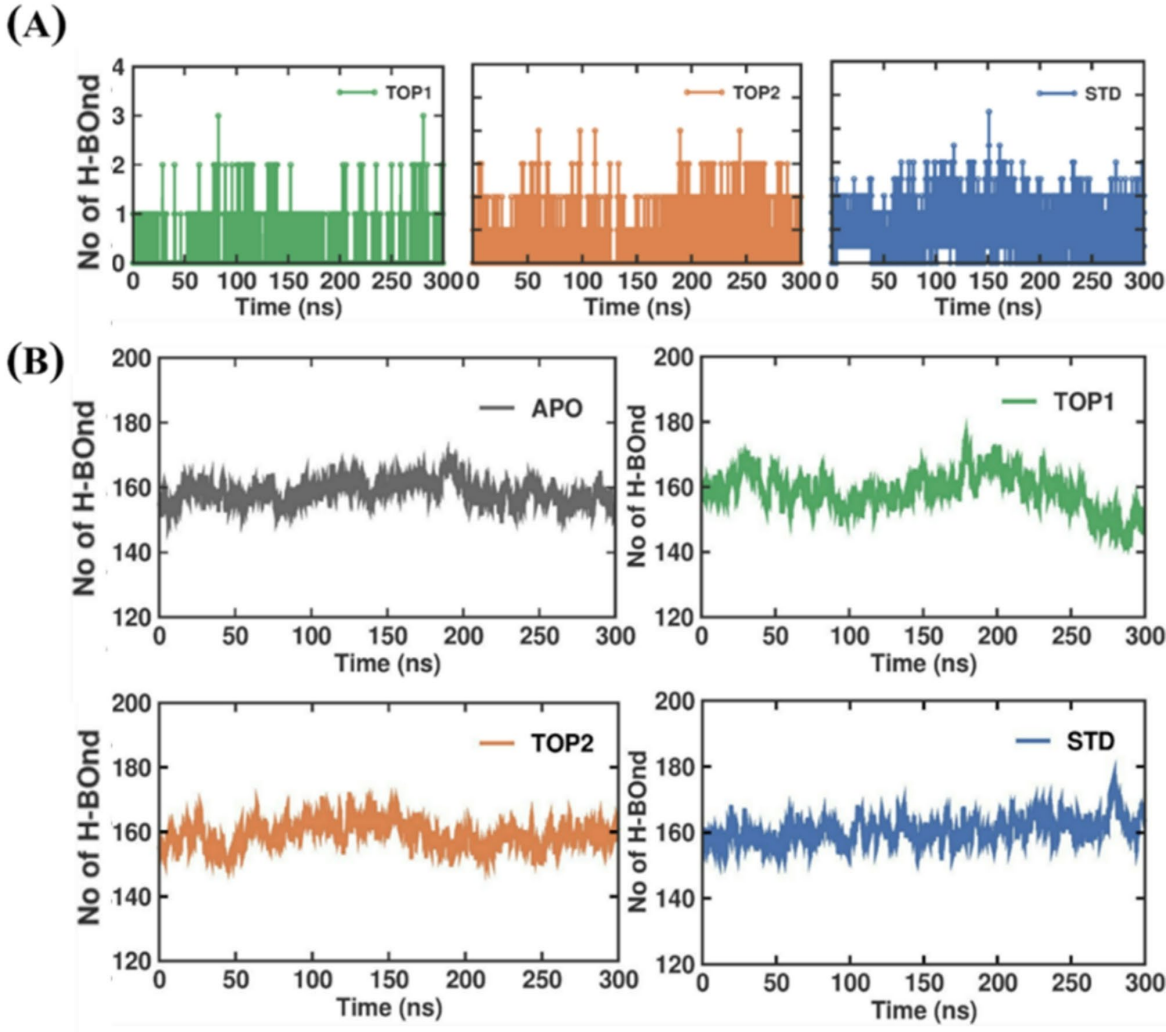
The H-bond interaction was analyzed in each ligand-bound complex through MDS. (**A**) The above plot depicts the inter-H bond of SND1 (APO), which is complex with TOP1, TOP2, and STD. (**B**) The plot below depicts the intra–H bond of SND1 (APO), which is also complex with TOP1, TOP2, and STD. Abbreviation: SND1 = Staphylococcal nuclease domain–containing protein 1; TOP1 = [4–(5,6,7,8–tetrahydro–4 H–cyclohepta[c][1,2]oxazol–3–yl)piperidin–1–yl]–[4–(trifluoromethyl)phenyl]methanone; TOP2 = 1–[2–hydroxy–2–(1–methylsulfonyl–3,4–dihydro–2 H–quinolin–6–yl)ethyl]–4(4–methylphenyl)piperidin–4–ol. Figures were generated by using QTGRACE Version 5.1.22.

The formation of hydrogen bonds between the ligand and protein makes the complex more stable; hence, to evaluate the stability of SND1–TOP1, SND1–TOP2, and SND1–STD complexes, a meticulous inspection of hydrogen bonds formed in each system during the MDS was considered. Besides stability, intermolecular (between SND1 and ligands) H–bonds could be attributed to the favorable free energy for binding ligands. The number of intermolecular H–bonds for each system is computed and is shown as plots in Fig. 6A. Based on the 300 ns trajectory analysis, the maximum number of H–bonds (5) was formed in the SND1–STD complex, while in the SND1–TOP2 complex, the maximum H–bonds involved in an interaction was 4. Compared to all complexes, the lower number of H–bonds (3) was in the SND1–TOP1 complex. Further, the H–bond occupancy analysis used the Visual Molecular Dynamics (VMD) package to determine the stable H–bonds formed between the SND1 and each ligand. Table S3 shows the H-bonds occupancy, where in SND1–STD, the protein forms 18 H–bonds, with the highest occupancy of Asn823 (2.03%) as a donor with STD. In the SND1–TOP1 complex, 10 H-bonds were formed between the active site amino acids and TOP1. Among them, Asn823, as a donor, exhibits the highest occupancy at 0.63%. Likewise, for SND1–TOP2, 11 H–bonds were formed, and more importantly, in this complex, more than one amino acid contributes to higher H–bond occupancy. As shown in Table S3, as a donor, the higher occupancy was seen in Thr688 (3.00%), followed by Gln792 (2.47%), Asn823, and Tyr766 with equal contributions of 1.27%, respectively. In addition to the inter-H bond, intra-H bond analysis of the SND1 protein is crucial as it helps us find its stability over a simulation period. Hence, we investigated the time-dependent behavior of intra-hydrogen bonds for APO, TOP1, TOP2, and STD complexes and plotted the results (Fig. 6B). The average intra–H–bond values for APO, TOP1, TOP2, and STD Complex were determined to be 158.62 ± 6.5 nm, 158.68 ± 8.05 nm, 159.19 ± 6.9 nm, and 160.66 ± 6.8 nm, respectively. Albeit no remarkable differences in the number of intra–molecular hydrogen bonds were observed, it is essential to note the fluctuations of hydrogen bonds formed from the start to the end of the simulation; compared to TOP1 and STD, the hydrogen bond formed remained stable in the TOP2–bound complex overall until 300 ns.

#### Binding free energy calculations

The Molecular Mechanics Poisson-Boltzmann Surface Area (MMPBSA) has become a more able and sturdy approach to authenticate protein-ligand interactions. The MMPBSA investigation scrutinized the binding affinity of SND1 interacting with STD, TOP1, and TOP2 throughout the MD trajectory. Post–simulation, the average binding free energy (ΔGbind) of SND1–STD, SND1–TOP1, and SND1–TOP2 was calculated across three different intervals: 150–200, 200–250, and 250–300 ns. The energy calculations derived at these three intervals are listed in Table 2. The calculated average free energy change of bonding (ΔGbind) for the SND1–TOP1 at all three-time intervals was found to be -95.271 ± 10.39567 kJ/mol, and for SND1–TOP2, the mean values were − 91.365 ± 18.939 kJ/mol, respectively. In contrast, the SND1–STD exhibited − 12.37 ± 14.498 kJ/mol. As suggested by the higher negative values, this result indicates that our identified putative drug candidates have a higher binding affinity than STD. The other concomitant energy terms, like van der Waals (vdW), electrostatic, and polar solvation energies, were also determined. The vdW energy of the complexes lies in the range of -47.619 ± 6.142 and − 190.741 ± 16.253, with the SND1–TOP2 exhibiting the maximum vdW energy (-189.589 ± 13.877). Similarly, the highest electrostatic energy was observed in the SND1–TOP complex, where the calculated electrostatic energy during 150–200 ns was − 36.864 ± 7.662 kJ/mol. Among all the complexes, SND1–TOP1 showed the lowest electrostatic energy (-15.950 ± 6.106 kJ/mol) when compared to TOP2 and SND1–STD (-32.263 ± 42.912 kJ/mol). For SASA energy, SND1–TOP2 demonstrated the highest negative energy (-21.564 ± 1.406 kJ/mol), followed by SND1–TOP1 (-13.272 ± 1.111 kJ/mol) and the least negative energy value from SND1–STD (-6.227 ± 1.564 kJ/mol). With the given ΔGbind values, the energy contribution of the active site residues for interacting with STD, TOP1, and TOP2 compounds was analyzed using the individual residue energy decomposition. From the molecular interaction investigations of the crystal structure 3OMC bound with R4me2S, we found that Phe686, Thr688, Glu689, Glu699, Val701, Glu702, Gly704, Glu708, Phe740, Asp742, Tyr746, Asn823 and Gln825 were involved in ligand binding. Hence, we sought to probe the role of these residues in binding with TOP1, TOP2, and SND1 by using an energy decomposition approach over three-time intervals: 150–200, 200–250, and 250–300 ns. In all the time intervals, as per the average predicted per residue binding energy, the SND1–TOP1 and SND1–TOP2 complexes exhibited similar mean values. For TOP1, the calculated average in 150–200 ns was (-0.82174), 200–250 (-0.87064), and 250–300 ns (-0.9031) kJ/mol, respectively. The average residue binding energy observed for SND1–TOP2 was 150–200: (-1.13475), 200–250: (-0.76333) and 250–300: (-0.48273). Among all the ligand–bound complexes, SND1–STD showed the least binding energy in all time intervals (150–200: -0.01372, 200–250: -0.00227, 250–300: 0.024092). The common active site residues considered crucial for ligand binding and contributed maximum energy in all complexes are Phe686, Val701, Asp742, Tyr746, and Gln825 (Table 2).

**Table 2:** MMPBSA values for the following protein–ligand complexes and energy calculation were predicted across three stable windows during simulations.

Name	Time intervals	Vander Wall energy (kJ/mol)	Electrostatic energy (kJ/mol)	Polar solvation energy (kJ/mol)	SASA (kJ/mol)	Binding energy (kJ/mol)
SND1–STD	150–200	-42.592 ± 12.932	-32.263 ± 42.912	69.289 ± 51.672	-6.227 ± 1.564	-11.793 ± 17.306
	200–250	-47.619 ± 6.142	-12.122 ± 6.210	49.037 ± 18.847	-6.227 ± 0.885	-16.931 ± 14.106
	250–300	-46.245 ± 4.523	-11.067 ± 9.565	55.271 ± 18.151	-6.345 ± 0.688	-8.386 ± 12.082
SND1–TOP1	150–200	-116.749 ± 8.825	-15.950 ± 6.106	44.268 ± 9.346	-13.272 ± 1.111	-101.703 ± 8.779
	200–250	-115.585 ± 9.423	-18.143 ± 6.866	49.260 ± 9.980	-13.338 ± 1.038	-97.806 ± 8.716
	250–300	-105.662 ± 14.394	-12.221 ± 8.337	44.224 ± 14.840	-12.645 ± 1.505	-86.304 ± 13.692
SND1–TOP2	150–200	-190.741 ± 16.253	-36.864 ± 7.662	157.449 ± 26.355	-20.419 ± 1.131	-90.575 ± 23.758
	200–250	-186.946 ± 19.306	-18.898 ± 9.002	132.005 ± 10.965	-20.369 ± 1.833	-94.209 ± 16.229
	250–300	-189.589 ± 13.877	-16.149 ± 9.969	138.936 ± 14.768	-21.564 ± 1.406	-88.365 ± 16.83

SASA = solvent accessible surface area; SND1 = Staphylococcal nuclease domain-containing protein 1; TOP1 = [4–(5,6,7,8–tetrahydro–4 H–cyclohepta[c][1,2]oxazol–3–yl)piperidin–1–yl]–[4–(trifluoromethyl)phenyl]methanone and TOP2 = 1–[2–hydroxy–2–(1–methylsulfonyl–3,4–dihydro–2 H–quinolin–6–yl)ethyl]–4–(4–methylphenyl)piperidin–4–ol.

#### Protein–ligand intermolecular interactions analysis

**Fig. 7:**
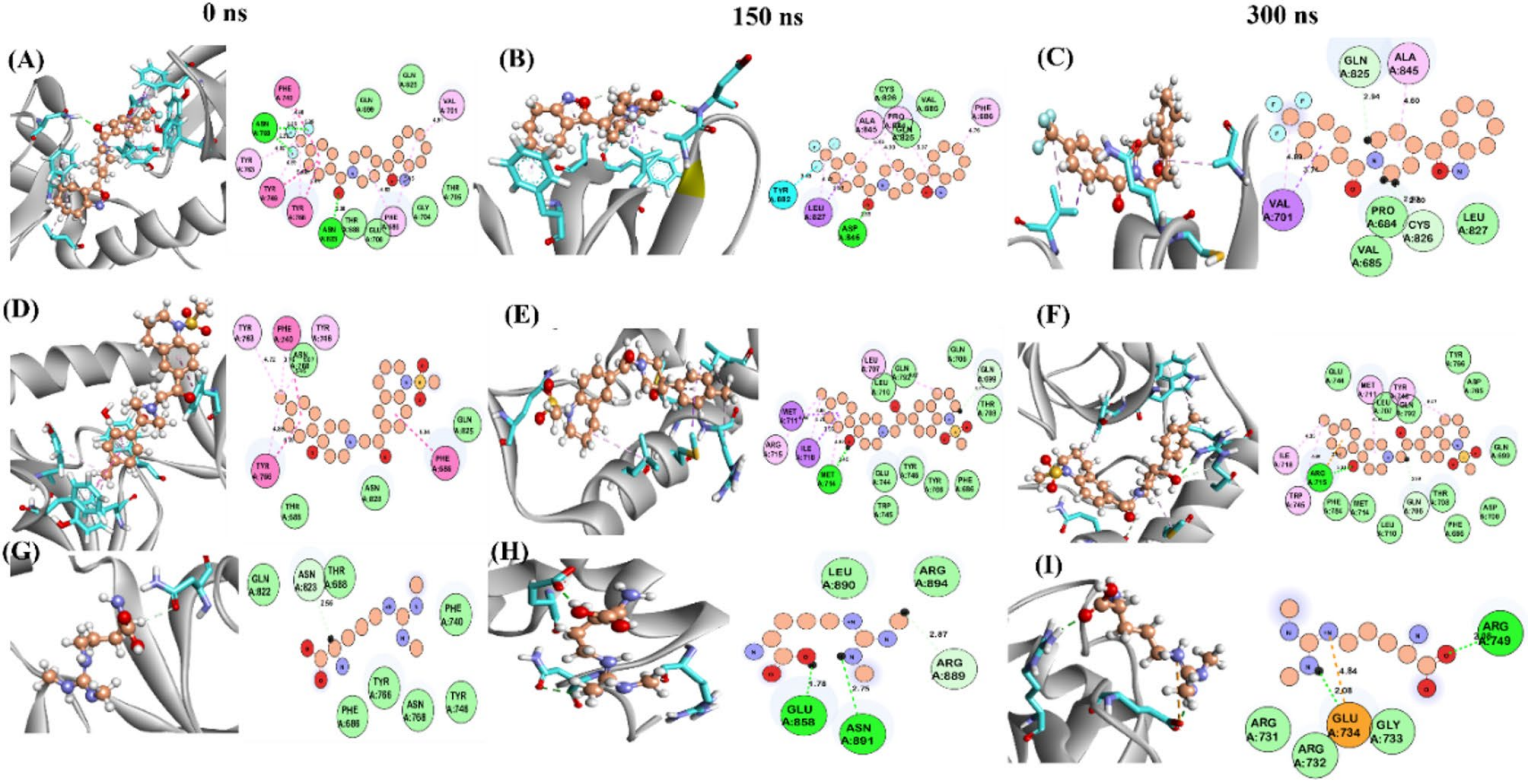
Molecular dynamics simulations of protein–ligand interactions of selected compounds represented as 3D and 2D diagrams at various times. (**A–C**) TOP1 binding with the SND1 active site. (**D–F**) TOP1 binding with the SND1 active site. (**G–I**) STD binding with the SND1 active site. Abbreviation: SND1 = Staphylococcal nuclease domain–containing protein 1; TOP1 = [4–(5,6,7,8–tetrahydro–4 H–cyclohepta[c][1,2]oxazol–3–yl)piperidin–1–yl]–[4–(trifluoromethyl)phenyl]methanone. Figures were generated by using Biovia Discovery Studio Version 2021.

The intermolecular interactions formed between protein and ligand are carefully analyzed to interrogate the discernment behind the efficiency of the binding energy of each ligand bound with the active site of SND1. The outcome of each ligand bound with the active site of SND1 during MDS trajectories is illustrated in Fig. 7. Three-time intervals 0, 150, and 200 ns time trajectories were taken, and the resulting intermolecular interactions and the distance between them are summarized as follows. The detailed interactions of each ligand-bound protein complex analyzed through MDS are summarized in Table S4. The TOP1 was found to be bound with the active site from 0 to 300 ns, and this was confirmed by the residues involved in earlier reported studies. The interactions with TOP1 form stable conventional and (C–H) hydrogen bonds, mainly with Asn768, Asn823, Gln825, and Cys826. The distance of these formed H–bonds was all within 3.0 Å. The extra interactions that stabilize the complex formation are halogen bonds where the oxygen atoms of Asn768 and Tyr682 establish halogen interactions with the fluorine atom of TOP1. In addition, the hydrophobic contacts such as π–π, π–σ, and π–alkyl further influence the complex stabilization. The active site aromatic amino acids like Phe740, Tyr746, and Tyr766 participate in π–type interactions and hydrophobic non-polar amino acids, viz. Val701 and Ala845, help in mediating alkyl type interaction with TOP1. Next, SND1–TOP2 complexes were investigated. Herein, two conventional H–bonds and 3 C–H–type hydrogen bonds were found. The residues that establish conventional H–bonds are Met714 and Arg715, while Gln699, Gln706, and Arg715 form a carbon–hydrogen bond with the TOP2. The functional groups involved in forming these interactions are listed in detail, as seen in Table S4. Similar to TOP1-SND1 binding, non-covalent interactions such as π–π, π–cation and π–alkyl interactions were present during the complex throughout the simulation period. The active site residues, Phe740, Trp745, Tyr746, Tyr763, and Tyr766, are responsible for π-alkyl interactions with TOP2, and Tyr746 forms stable π–alkyl interaction as it exists present till at 300 ns. The intermolecular interaction analysis of reference compound STD bound with SND1 shows that three conventional H–bonds were formed. The oxygen atom in Glu858, Glu734, and Arg749 helps mediate the H–bond with the hydrogen atom of the STD. It is also noteworthy that no halogen or π–π, π–cation, and π–alkyl interactions were present in the SND1–STD complex. Furthermore, in between the TOP1 and TOP2–bound protein complexes, the distance of H–bond form at 300 ns in the TOP2 (2.3 Å) is much lesser than TOP1 (2.9 Å), which signifies the tight interaction of TOP2 binding with SND1.

#### Secondary structure analysis

**Fig. 8:**
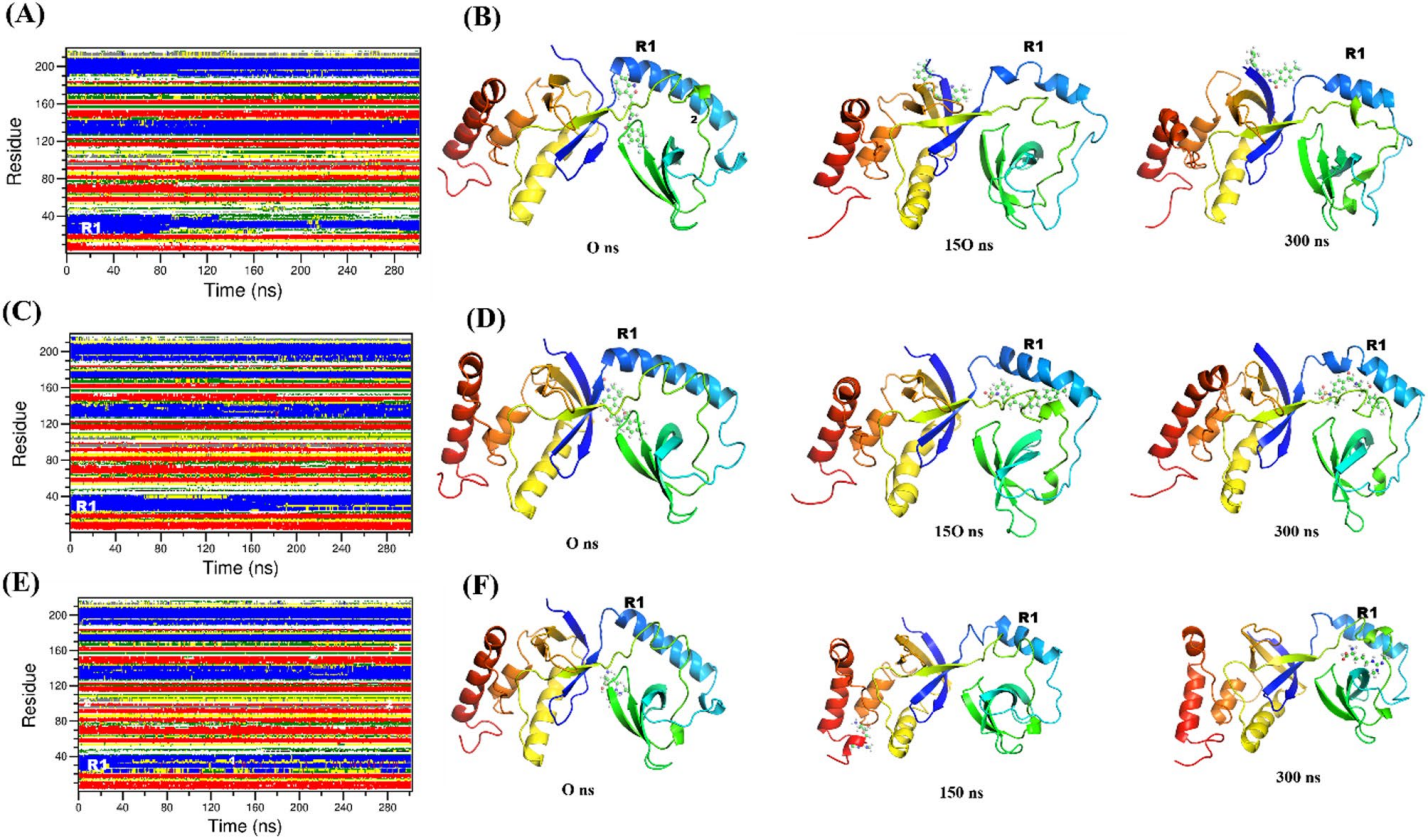
Secondary structure evolution of ligand-bound protein complexes (**A**) Time evolution of secondary structural units in TOP1–SND1 complex are shown (**B**) Structural snapshots showing the evolution of secondary structure changes in TOP1–bound protein at 0, 150, and 300 ns. (**C**) Time evolution of secondary structural units in TOP2–SND1 complex are shown (**D**) Structural snapshots showing the evolution of secondary structure changes in TOP2–bound protein at 0, 150, and 300 ns. (**E**) Time evolution of secondary structural units in STD–SND1 complex are shown (**F**) Structural snapshots showing the evolution of secondary structure changes in STD–bound protein at 0, 150, and 300 ns. Abbreviation: SND1 = Staphylococcal nuclease domain-containing protein 1; TOP1 = [4–(5,6,7,8–tetrahydro–4 H–cyclohepta[c][1,2]oxazol–3–yl)piperidin–1–yl]–[4–(trifluoromethyl)phenyl]methanone; TOP2 = 1–[2–hydroxy–2–(1–methylsulfonyl–3,4–dihydro–2 H–quinolin–6–yl)ethyl]–4–(4–methylphenyl)piperidin–4–ol. Figures were generated by using PyMol version 4.6.0.

The Define Secondary Structure of Protein (DSSP) tool determined the secondary structure variations when SND1 binds with ligands. This tool primarily designates la-bels to protein amino acids based on diverse geometrical properties and H–bonding arrangement. By employing the do dssp command in the GROningen MAchine for Chemical Simulations (GROMACS), the secondary structure evolution over the entire simulation time for SND1–TOP1, SND1–TOP2, and SND1–STD complexes was examined. Trivial changes were noted in α-helices, β–sheets, and turns in SND1–TOP1 across different periods up to 300 ns. The changes corresponding to these regions are displayed as snapshots. Three distinct time intervals, 0, 150, and 300 ns, were chosen to represent these changes in all the ligand–bound protein complexes (Fig. 8). The residues from 701 to 720, labeled as one corresponding to α-helices, were found to be changed into a loop at 150 and 300 ns in SND1–TOP1. Likewise, the loop region (780–790) was found to be changed into a bend after 230 ns. Similar loop region changes were noted in the SND1–TOP2 complex as well. In the SND1–STD complex, the distortion is mainly found in α-helix (710–720) and loop (850–855) regions. These regions exhibit more flexibility, as evident with the RMSF values, and significantly, these structural movements have not affected the ligand binding. This shows that the secondary structural changes are minor and possibly underwent conformational changes to adopt the TOP1 and TOP2 be bound with the active site throughout the evolution of up to 300 ns.

#### Cavity analysis

Cavity analysis plays a crucial role in understanding the functional properties of proteins. Researchers gain insights into ligand binding sites, enzymatic activities, and protein-protein interactions by identifying and characterizing pockets and clefts within protein structures. The cavity analysis was performed using the CASTp webserver (http://sts.bioe.uic.edu/castp/index.html?1ycs). As aforementioned, we could deduce a distinct change in the active site secondary structure in the TOP1–bound SND1 complex. To further probe the active site changes, we measured each system’s inhibitor/ligand bound cavity volumes to understand the dynamics of bound inhibitor conformational changes upon binding. Table 3 provides quantitative data on the surface area (SA) and volume of SND1 proteins under different conditions (APO, TOP1, TOP2, STD) at various ns values (0, 100, 200, 300). In the APO state, SND1 exhibits an initial surface area of 154.087 Å² and volume of 62.728 Å³ at 0 ns, decreasing to 104.974 Å² and 31.741 Å³ at 100 ns, then increasing to 119.105 Å² and 166.86 Å³ at 200 ns before further rising to 147.281 Å² and 99.868 Å³ at 300 ns. In the SND1-TOP1 state, surface area and volume start at 119.625 Å² and 117.675 Å³ respectively, at 0 ns, then decrease drastically to 45.44 Å² and 158.655 Å³ at 100 ns, followed by notable fluctuations reaching 348.276 Å² and 284.357 Å³ at 200 ns, before stabilizing at 182.988 Å² and 197.754 Å³ at 300 ns. SND1–TOP2 exhibits variations starting from 111.301 Å² and 44.947 Å³ at 0 ns, then rising to 183.505 Å² and 300.033 Å³ at 100 ns, followed by fluctuations and eventual stabilization at 106.829 Å² and 72.43 Å³ at 300 ns. In the SND1–STD state, surface area and volume begin at 76.784 Å² and 65.124 Å³ respectively at 0 ns, increase to 198.585 Å² and 368.33 Å³ at 100 ns, then fluctuate around 104 Å² and 93 Å³ at 200 ns, before stabilizing again at 171.454 Å² and 131.712 Å³ at 300 ns. Structural snapshots that depict the cavity in three different ligand-bound complex is shown in Figure S2.

**Table 3:** The table summarizes the cavity volume of each ligand-bound and non–bound complex.

Name	Time (ns)	Area (SA) Å2	Volume (SA) Å3
SND1–APO	0	154.09	62.73
	100	104.97	31.74
	200	119.10	166.86
	300	147.28	99.87
SND1–TOP1	0	119.62	117.68
	100	45.44	158.66
	200	348.28	284.36
	300	182.99	197.75
SND1–TOP2	0	111.30	44.95
	100	183.50	300.03
	200	116.99	89.56
	300	106.83	72.43
SND1–STD	0	76.78	65.12
	100	198.58	368.33
	200	104.92	93.35
	300	171.45	131.71

SND1 = Staphylococcal nuclease domain-containing protein 1; TOP1 = [4–(5,6,7,8–tetrahydro–4 H–cyclohepta[c][1,2]oxazol–3–yl)piperidin–1–yl]–[4–(trifluoromethyl)phenyl]methanone; TOP2 = 1–[2–hydroxy–2–(1–methylsulfonyl–3,4–dihydro–2 H–quinolin–6–yl)ethyl]–4–(4–methylphenyl)piperidin–4–ol.

#### Principal component and free energy landscape analysis

**Fig. 9:**
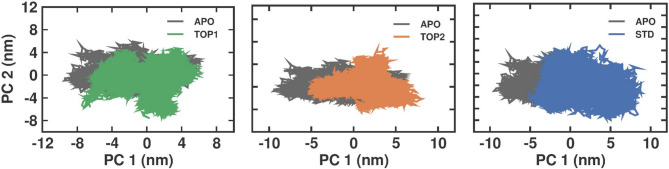
The principal component analysis 2D projection plot shows the conformation sampling of APO, TOP1, TOP2, and STD on PC1 and PC2. Abbreviation: SND1 = Staphylococcal nuclease domain-containing protein 1; TOP1 = [4–(5,6,7,8–tetrahydro–4 H–cyclohepta[c][1,2]oxazol–3–yl)piperidin–1–yl]–[4–(trifluoromethyl)phenyl]methanone; TOP2 = 1–[2–hydroxy–2–(1–methylsulfonyl–3,4–dihydro–2 H–quinolin–6–yl)ethyl]–4–(4–methylphenyl)piperidin–4–ol. Figures were generated by using Qt-based Grace (QTGRACE) Version 5.1.22.

**Fig. 10:**
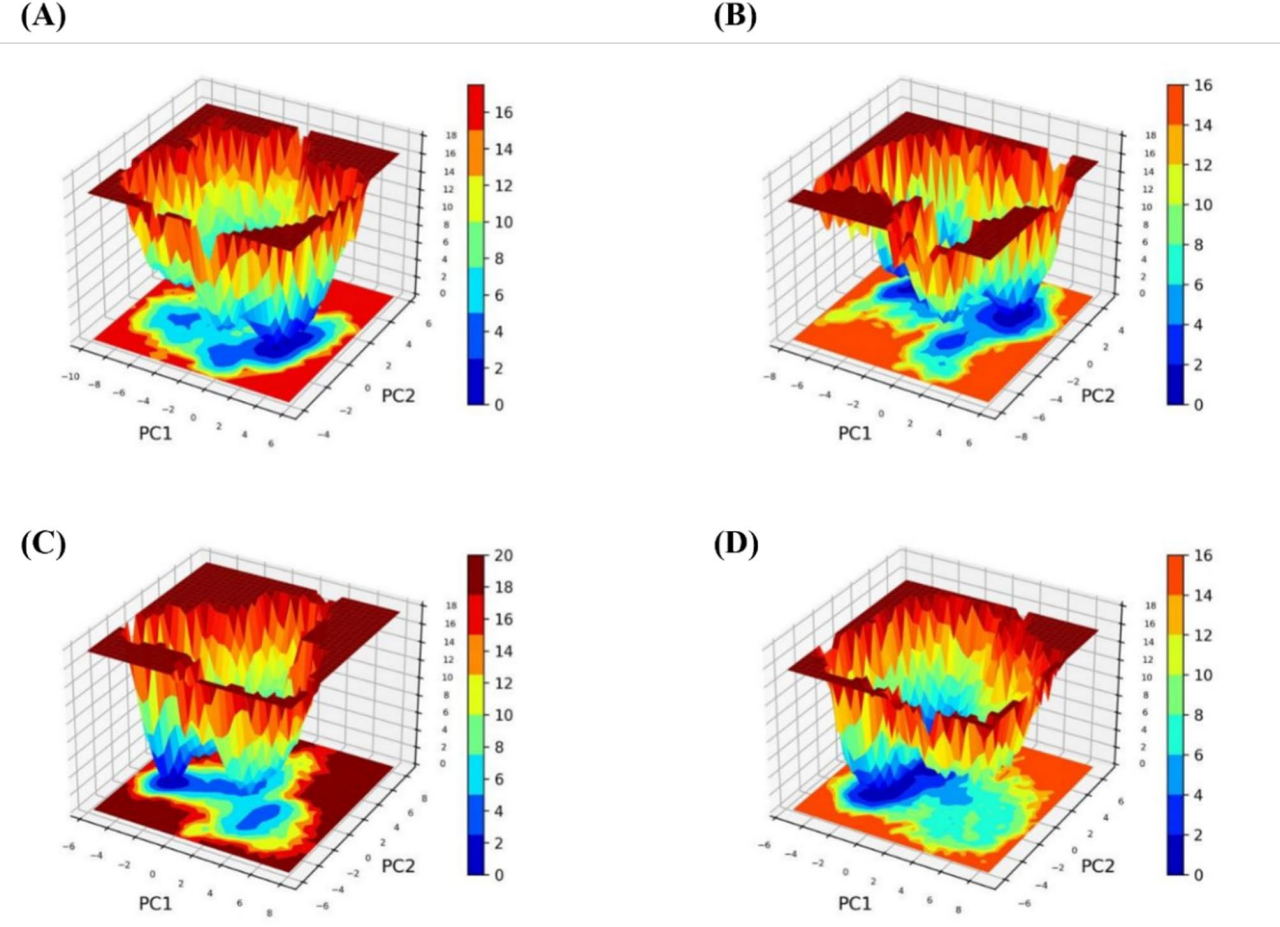
The free energy landscape for 3D and 2D represents the Gibbs free energy landscape as a function of PC1 and PC2. The coloring pattern shows the energy distribution: Blue indicates the conformational space with minimum energy (stable state), while red indicates a conformational space with maximum energy (unstable state). (**A**) APO form. (**B**) SND1–TOP1. (**C**) SND1–TOP2 and (**D**) SND1–ST. Abbreviation: SND1 = Staphylococcal nuclease domain–containing protein 1; TOP1 = [4–(5,6,7,8–tetrahydro–4 H–cyclohepta[c][1,2]oxazol–3–yl)piperidin–1–yl]–[4–(trifluoromethyl)phenyl]methanone; TOP2 = 1–[2–hydroxy–2–(1–methylsulfonyl–3,4–dihydro–2 H–quinolin–6–yl)ethyl]–4–(4–methylphenyl)piperidin–4–ol. Figures were generated by using GraphPad Prism Version 8.3.0.

The Principal Component Analysis (PCA) is used to unveil the changes in the dynamic pattern between APO and the protein-ligand bound complexes. The primary component in the covariance matrix was made utilizing its cognate eigenvectors, using the changes in the trajectories that lead to projection changes. The simulated trajectories were projected as PC1 and PC2, and the matrix was generated through molecules’ atomic eigenvectors, leading to the display of protein-ligand complex structural variations. The protein dynamics that occurred are displayed in Fig. 9. Our findings reveal that SND1–TOP1 and SND1–TOP2 complexes had less phase space than SND1–STD. The total flexibility of the apo and each ligand-bound protein complex is assessed using the diagonalized covariance matrix of the changes in the Cα-atom position. It is noticed that the observed flexibility of SND1–TOP1, SND1–TOP2, and SND1–STD are respectively. Furthermore, the Gibbs energy landscape was analyzed with the help of PC1 and PC2 eigenvectors obtained for the apo and bound complexes. The Gibbs free energy denotes the formed complexes’ conformation states by showing the regions colored from blue to red. The stable confirmation, which possesses the lowest energy, is represented in dark blue, while the unstable conformations having higher energy are depicted as dark red. The yellow indicates intermediate unfavorable con-formations, as shown in 2D and 3D plots. From the 3D plots, it is clear that the SND1–TOP2 complex shows dark blue regions with deeper intensity and lower energy values, which indicates good stability. In the SND1–TOP1 complex, more intermediate conformations are present, and stable conformations seem to be broader, as is evident with a single dark blue region. This shows that TOP2 binding with SND1 makes the complex less stable when compared to TOP1 binding. On the other hand, the SND1–STD displays very few intermediate energy minima and steep, narrow lower energy dark blue regions, which signifies better stability Fig. 10.

## Discussion

SND1 protein is often misregulated in HCC, behaves as an oncogene, and drives tumor growth in the liver^21^. Our initial findings with the CGSA database reveal that the SND1 is mutable, and around 1.3% of somatic mutation was recorded in HCC patients. In addition, SND1 gene expression (mRNA level) overexpression was observed and directly linked with the various implications for HCC–affected patients^25^. Lately, the mounted evidence shows that the SDMA marks created by the PRMT5 also play a vital role in HCC, and these SDMA marks are generally read by the SND1 protein and eventually regulate various molecular signaling events that promote HCC^20^. Owing to such high significance, we thought SND1 would be an excellent therapeutic target for HCC. The SDMA binding pocket of SND1 is chosen as our study target to develop novel inhibitors that could prevent the SND1 from reading SDMA marks after binding with our putative inhibitors. Notably, no commercially available drug or inhibitors could target the SND1 protein and avoid SND1–PRMT5 interaction. This urged us to identify and develop novel inhibitors with high specificity and less toxicity. To accomplish that, we explored the ASINEX database and selected the “ELITE” library, which comprises 91,001 compounds that could potentially bind with the SND1 active site.

This study employed molecular docking, pharmacokinetic properties prediction, toxicology analysis, MD simulation, and MMPBSA methods to discover the best inhibitors against SND1. Autodock Vina was utilized for the virtual screening of compounds from the chosen library, and docking was performed against the active site of the SND1. The docking predicted the compounds that have the best affinity with the protein, and based on the docking score, the top 15 compounds were selected. As depicted in Table S5, the docking score for the top 100 compounds ranged from − 10.4 to -9.6 kcal/mol, which is better than the reference compound SDMA’s binding score of -4.6 kcal/mol. A better binding affinity (1–fold higher) was observed in our identified compounds. After docking, the top 15 compounds that show better binding affinity than reference inhibitor SDMA were taken and subjected to hierarchical clustering and pharmacokinetic analysis. Based on the results, we found that the top 2 best docking score compounds, TOP1 and TOP2, possess good pharmacokinetic profiles with no significant toxicity observed in them. As per the prediction, TOP1 and TOP2 were non-mutagenic and non-hepatoxic and showed no abnormal effects on reproductive receptors and stress response pathway proteins. The best molecular docking pose of TOP1, TOP2, and SDMA were further subjected to MDS, as they represented highly selective inhibitors for SND1. Next, the MDS analysis and free energy calculations using MM/PBSA corroborate well with predicted docking scores. The results are depicted as RMSD, RMSF, Rg, SASA plots, and the number of intermolecular interactions formed during the MDS for each complex. RMSD of all each-ligand bound complex was stable and balanced during the 300 ns period. The RMSD distribution values plotted as probability distribution function (PDF) indicate the stable binding of TOP1 and TOP2 with higher probability over SDMA. In particular, the RMSD PDF plot for TOP2 shows a single peak and lower SASA concerning PDF, suggesting that the TOP2–bound SND1 complex is more stable and intact than the SND1–TOP1 complex (Figure S4). Further RMSF analysis shows higher fluctuation in the residues between 710 and 720, noted in the TOP1–bound protein complex. As depicted in Fig. 2, compared to Apo, ~ 0.1 to 0.3 nm fluctuation was observed upon TOP1 binding, while TOP2 does not influence any significant deviations in RMSF values. These highly fluctuating regions are essential as they correspond to the α–helix, which links Tudor and SN–like domains. Besides acting as a connecting bridge, the amino acids present in this α–helix (linker) also act as an active site, where the amino acids such as Gly704 and Glu708 were found to have interacted with the R4me2s (SDMA region of the PIWIL1 peptide, SND1-PIWIL1–protein protein interaction). Thus, the higher flexible movements of the residues in this region might be attributed to the changes in protein secondary structure and help in ligand movement from the active site (Figure S5). The DSSP structure analysis further confirms our assumption, where, as shown in Fig. 7, at 0, 100, and 300 ns trajectories, the TOP1, which initially bound in the Tudor domain active site, was moving away at 150 ns from its initial binding site and eventually at the end of 300 ns, the TOP1 was found to be bound with the active site residues of the α–helix region. As aforementioned, the Tudor domain and linker α–helix constitute the active site. Although local dynamics in the TOP1 ligand were noticed, it neither drifted away nor lost its binding with the SND1 active site. In contrast, no movement or displacement was observed in TOP2 binding in the SND1 active site. DSSP plots and structural snapshots show no significant loss in the α–helix region, which was convinced with the RMSF analysis well, where, unlike TOP1, TOP2 had not exerted severe flexibility compared to APO protein. The reference ligand SND1–STD illustrates modest changes in the α–helix region, and these conformation changes result in the movement of STD within the active site.

**Fig. 11:**
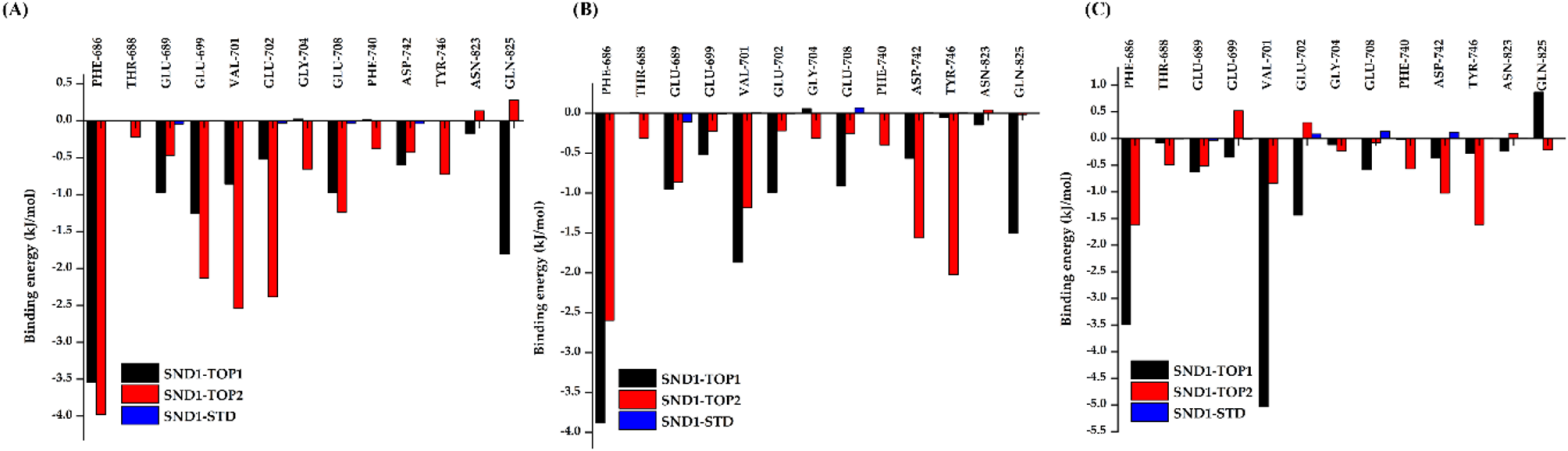
Bar chart showing the residue per binding energy across TOP1, TOP2, and STD–bound SND1 complexes: (**A**) 0 ns, (**B**) 150 ns, and (**C**) 300 ns. Abbreviation: SND1 = Staphylococcal nuclease domain-containing protein 1; TOP1 = [4–(5,6,7,8–tetrahydro–4 H–cyclohepta[c][1,2]oxazol–3–yl)piperidin–1–yl]–[4–(trifluoromethyl)phenyl]methanone; TOP2 = 1–[2–hydroxy–2–(1–methylsulfonyl–3,4–dihydro–2 H–quinolin–6–yl)ethyl]–4–(4–methylphenyl)piperidin–4–ol. Figures were generated by using OriginPro 8.5.

The initial findings on ligand–induced protein secondary changes have enticed us to find more profound insights into intermolecular interaction differences with SND1–TOP1, SND1–TOP2, and SND1–STD complexes. Among various intermolecular interactions, H–bond interaction between ligand and protein is of utmost importance as they are considered more stable. With such insight, a detailed investigation of H–bond interacting amino acids for these complexes was performed, and the results concluded that TOP2 and STD form conventional H–bond interactions throughout the simulation time. In contrast, the TOP1, which initially had a conventional H–bond with the SND1, was found to be missing during 300 ns time. For the TOP1–SND1 complex, Asp846 forms an H–bond at 150 ns, and when the simulation reaches 300 ns, the Asp846 loses its interaction, and no active site amino acids from the Tudor domain and α–helix region form an H–bonding with TOP1. However, in the SND1–TOP2 complex, Met714 at 150 ns and Arg715 at 300 ns from the α–helix region form a conventional H–bond, which helps the TOP2 to be bound with the active site. Furthermore, at 300 ns, the Trp745 and Tyr746 from the Tudor domain establish the π–alkyl interactions with the TOP2. Contrarily, such interactions from the Tudor domain were absent in the SND1–TOP1 complex during the end of the simulation. It is well known that π–type interactions are crucial for the efficient functioning of membrane proteins and ion channel receptors. For example, cation– π interaction plays a vital role in the Ach receptors in the brain to bind with nicotine. Besides the intermolecular analysis, probing the energy per residue decomposition analysis for each complex affirms that TOP2 binding was more stable and specific than TOP1 and STD. This is because the calculated average binding energy for the critical active site amino acids that led to interactions, viz. Phe740, Asp742, Tyr746, and Gln825, for TOP1 binding was − 0.0505, for TOP2: -0.85538, and for STD: +0.0317, respectively (Fig. 11). As discussed earlier, these amino acids form the active site of the Tudor and α-helix region, which is responsible for recognizing the SDMA marks and the average individual binding energy for individual essential amino acids determined at 150–220, 200–250, and 250–300 ns elucidates the TOP–bound protein complex (-0.85538 kcal/mol) is more stable and bound with good affinity than TOP1 and STD. With such proven evidence on docking score, intermolecular interactions, and residue per binding energy, we then sought to analyze the active site space of each ligand–bound complex (Figure S3). By measuring the area and the volume of the active site cavity where the ligand binding occurs, we found that TOP2 binding has not markedly affected the active site. We found that the cavity area size and volume size did not change significantly when compared to the APO form (surface: 131.3618 Å, volume 90.29925 Å), where the average area size for SND1–TOP2 is measured to be 129.658 (Å), and the volume of the cavity is 126.7435 (Å). On the other hand, significant differences in cavity area and volume were observed upon TOP1–binding, where the calculated average surface was 174.082 Å and volume 189.603 Å, respectively. STD binding, albeit, did not exhibit huge differences like TOP1, but when compared to APO and TOP2–bound complex, the average values were higher (surface: 137.9365 Å, volume 164.629 Å). This further confirms why the RMSD, RMSF, Rg, and SASA for the SND1–TOP1 complex are more remarkable than TOP2. The TOP1, after binding with the active site, has induced some conformational changes, mainly with the α-helix region. The movement of the flexible loop region resulted in a bigger active site cavity, which led the TOP1 to glide in the active site. Finally, at the end of the simulation time (300 ns), the binding was observed at the end of the active site region. However, such displacement was not observed with TOP2 binding, where the TOP2 was intact and could maintain interactions with both the Tudor domain and α–helix region. Thus, we believe that TOP2–bound SND1, undergoing specific conformational changes, could not help itself interact with PRMT5 to read the SDMA marks, as conformational changes will lead to disorientation. However, further in vitro and in vivo experiments are necessary to validate our hypothesis. Still, we believe this study might be a new gateway and an interesting therapeutic one for managing HCC.

## Materials and methods

### SND1 gene expression and survival analysis

The SND1 gene mutation in HCC was analyzed using the Gene Set Cancer Analysis (GSCA) database. This portal is an invaluable tool for exploring the mutations occurring in the various oncogenes, identifying protein-protein interactions, etc. SND1 mutation in HCC was predicted. Further, the proteins interacting with SND1 are mapped, and the protein-protein interactions are shown as clusters. The GSCA server analyzed SND1 gene expression in HCC patients by predicting the gene expression fold change through mean (normal)/mean (tumor) by examining the p–value (Student’s t–test), normalized with the false discovery rate (FDR). In addition, the SND1 expression in LIHC was determined using the UALCAN web server (http://ualcan.path.uab.edu/index.html). This portal, utilizing the TCGA and CPTAC OMICS data, helps to analyze the correlation between SND1 expression in LIHC and overall survival using the datasets of HCC tumor samples.

### Protein preparation and virtual screening

The SND1 protein structure was downloaded from the Research Collaboratory for Structural Bioinformatics Protein Data Bank (RCSB PDB) with the corresponding PDB ID 3OMC. The crystal structure 3OMC is a human SND1 extended Tudor domain complexed with symmetrically demethylated arginine PIWIL peptide R4me2S^26^. The water molecules present in the complex were removed using Biovia Discovery Studio Visualizer, and the resulting protein structure was processed to initiate docking procedures. For example, the hydrogen atoms were added, the bond order was assigned, and the missing atoms and residues, if present, were filled. The protonation state of Asp, Glu, and His residues was also completed. On the other hand, the virtual screening was done using the ASINEX database, which contains an “Elite” library of 91,101 compounds. This study harnessed this public database to screen the hit molecules that could be SND1 inhibitors. The structures of these compounds were saved in Structure-Data File (SDF) format and were subsequently used for further processing. All ligands used in the study were downloaded as a single .sdf file from the ASINEX database. Subsequently, this file was used for energy minimization, employing the MMFF94 force field along with the conjugate gradient optimization algorithm for 200 steps in PyRx before starting the docking process.

### Molecular docking analysis

All 91,001 compounds attained from the ASINEX database were employed for docking studies to confine the number of potential hits from the virtual screening. Firstly, the Open Babel Software 3–1–1 was used to convert the SDF format of each ligand to 3D PDB format. AutoDock Vina was employed, the grid box was placed in the active site of the SND1 protein, and the docking process was initiated. Lamarckian Genetic Algorithm was selected, and 10 poses were generated. During the docking, the SND1 structure was kept rigid while all the ligand’s binding modes were maintained to be rotated and flexible during the binding. In the post-docking procedure, the output docking poses were analyzed to predict their binding affinity and the best poses were selected. The ligands with better docking scores (kcal/mol) values were ranked accordingly, and the STD docking score was chosen as a reference. A total of 15 ligands were found to have the best docking score and were ranked in the order that lies from − 10.4 to -9.5. All 15 ligands (compounds) interactions with the SND1 active site were assessed using the Discovery Studio visualizer and Pymol. The docking and results interpretation are done using previously described methods^23^.

### Drug-likeness, pharmacokinetic properties, and biological activity of compounds

The calculation of physicochemical properties for STD (PubChem ID: 169148), TOP1 (PubChem ID: 92413470), and TOP2 (PubChem ID: 46954798) was accomplished using Swiss–ADME software (http://www.swissadme.ch/, accessed on 10 September 2024). The drug–likeness properties referred to as Lipinski’s rule of five, which include molecular weight, H–bond acceptors, H–bond donors, and logP values, are determined through Swiss-ADME. Prediction of Lipinski’s rule of five for virtually screened compounds is of utmost importance as the compounds that meet the criteria could have good folding ability and exert better therapeutic effects. Next, ADMET 2.0 (http://admetmesh.scbdd.com/, accessed on 10 September 2024) was employed to estimate various pharmacokinetic properties like cytochrome P450, gastrointestinal absorption, and excretion. Likewise, the prediction of rat oral toxicity was analyzed using the PROTOX–II web server (http://tox.charite.de/protox2.com/, accessed on 10 September 2024). This web server helps to evaluate the half–maximal lethal doses (LD_50_ in mg/kg) based on the similarity of the compound. Further, the Prediction of Activity Spectra of Substances (PASS) (https://genexplain.com/pass/, accessed on 10 September 2024) was utilized to predict the biological activity of TOP1 and TOP2. PASS, exhibiting an average prediction accuracy of ~ 95% based on leave–one–out cross–validation (LOO CV) estimation, utilizes a Bayesian approach to determine the probable biological activity of the compounds based on their structure. A similarity search of the target compounds was done through DrugBank to predict biological activity using closely related structural analogs.

### Molecular dynamics simulations

Molecular dynamics (MD) simulation, a computational technique employing Newton’s laws of motion, was used to look at the stability of the docked protein-ligand complexes. Considering the best docking scores of each compound attained, the SND1–TOP1, SND1–TOP2, and SND1–STD were subsequently applied for the simulation studies to disseminate their interaction stability for 300 ns. The simulation was performed using the GROMACS software package-2019.4, a widely used and well–established MD simulation software (http://www.gromacs.org/), and a CHARMM36 force field^24^. The process begins with the protein–ligand minimization in a vacuum via the steepest descent algorithm and minimizing the system’s potential energy by appropriately placing the complex’s atomic coordinates. Once the depreciation was completed, the Simple Point Charge (SPC) water model was applied to solve the complex. The SPC water model is a simple model representing water molecules and is often used as a starting point for more complex water models. The gmxgenion tool was utilized to electro-neutralize the system. Further, energy minimization was carried out to eliminate steric clashes and structure optimization. After energy minimization, the system equilibration was achieved in two steps. Initially, for 100 picoseconds of NVT equilibration, the system was heated up to 310 K to stabilize the temperature of the system. Followingly, in the second step, during 100 picoseconds of NPT ensemble, the pressure and density of the system were stabilized. The temperature at 310 K and pressure at 1 bar are used for the NPT ensemble process. The resultant structure from the NPT equilibration phase was subsequently used for a final production run for 300 ns simulation time. The NPT ensemble simulates systems at constant temperature and pressure, which is commonly encountered in biological systems. Finally, the docked protein-ligand complexes’ stability for TOP1, TOP2, and STD was evaluated by using the attained trajectory and tools provided by the GROMACS software package, including the protein RMSD, RMSF, RG, SASA, H–Bond, and free energy landscape analysis^23^. The data obtained were analyzed using PyMol, Discovery Studio 2021, and VMD for figure generation. The XmGrace and OriginPro 8.1 tools were utilized for graph plotting.

### MMPBSA analysis

The MMPBSA is a potential method to cross–validate and affirm the docking score predicted in Autodock Vina. Herein, the MMPBSA estimations were carried out for the SND1–TOP1, SND1–TOP2, and SND1–STD complexes. The 150–200, 200–250, and 250–300 ns of Gromacs trajectories for each complex were used for the ΔG calculation. The MMPBSA calculation was performed using the g_MMPBSA program. This program utilizes MmPbSaStat.py, a Python script (Python version 3.10.5) for average energy prediction. Further, the average of the energy contribution of each residue was attained by using the script MmPbSaDecomp.py (https://rashmikumari.github.io/g_mmpbsa/Download-and-Installation.html). The energy decomposition for each complex over the specified ns of the trajectory was utilized to predict the binding affinity and the other factors resulting in various energy terms to the complete binding energy. The ΔG was estimated as per the equation below.

1 $$\Delta {\text{Gbinding}}\,=\,{\text{Gcomplex}}-({\text{Gprotein}}\,+\,{\text{Gligand}})$$

Gcomplex means the free energy of the protein-ligand complex (PL denotes the used ligand), and Gprotein and Gligand denote the protein and ligand-free energies.

The estimation of free energy in bound and unbound form is done by.

2 $${\text{Gx }}={\text{ }}\left( {{\text{EMM}}} \right)-{\text{TS }}+{\text{ }}\left( {{\text{Gsolv}}} \right)$$

X corresponds to the PL complex or free form, P or L. EMM determines the average molecular mechanics, TS refers to the entropic contribution, and Gsolv denotes the free energy solvation of protein-bound with the ligand.

The molecular mechanics (EMM) was calculated by considering the electrostatic and van der Waal’s interactions between the protein–ligand complex, as shown in Eq. (3). Gsolv means the linear Poisson–Boltzmann equation for individual states (Gpolar), and solvent-accessible surface area was estimated through non-hydrophobic interactions.

3 $${\text{EMM}}\,=\,{\text{Ebonded}}\,+\,{\text{Enon}}-{\text{bonded}}$$

4 $${\text{Gsolv}}\,=\,{\text{Gnonpolar}}\,+\,{\text{Gpolar}}$$

To know the quantitative amount of energy contribution of the desired active site interacting amino acids with the inhibitors, the per–residue decomposition analysis was performed. To evaluate the individual residues that lie within 6Å of the SND1 active site contribution in terms of the free energy of binding, snapshots at three different periods (0 ns, 150 ns, and 300 ns) were selected. Phe686, Thr688, Glu689, Glu699, Val701, Glu702, Phe740, Asp742, Tyr746, Asn823 and Gln825 are the active site amino acids that play a crucial role in forming interactions with the inhibitors. Hence, these amino acids were considered and the g MMPBSA tool was employed to predict the binding energies from the MMPBSA run. Followingly, the “MmPbsaDecomp.py” script was used for determining the overall binding energy contributed from each targeted individual amino acid.

## Conclusions

In this study, SND1, one of the highly upregulated proteins affecting the patient’s survival during liver carcinoma, has been taken as our inhibitor target. As no drugs are available to inhibit SND1, which drives the HCC, we successfully harnessed the virtual screening approach to identify novel compounds from the ASINEX library. The potential hits exhibiting the best affinity with the SND1 active site were ranked based on the binding score obtained through AutoDock Vina. The top two ranked compounds showed promising ADMET properties and cleared the false–positive prediction during the PAINS analysis. Further, the molecular dynamics simulations and MMPBSA evaluation indicated that TOP1 and TOP2 have higher binding affinities when compared to the reference compound SDMA. Notably, the RMSD, RMSF, Rg, and investigation of the intermolecular interactions of the TOP1 and TOP2–bound protein complex reveal that SND1–TOP2 has more robust and more stable interactions with the SND1 active site. The MMPBSA and residue per energy decomposition analysis firmly defined the reason behind the stability and strength of the TOP2 binding. In summary, the TOP2 compound was found to have promising potential and can be further evaluated as an SND1 inhibitor with the in vitro experimental results.

## Supplementary Information

Below is the link to the electronic supplementary material.

Supplementary Material 1

## Data Availability

The data presented in this study are available on request from the corresponding author.

## Abbreviations

ADMA: Asymmetric dimethyl arginine
5-Aza: 5-Azacytidine
CYP: Cytochrome P-450
DNMT1: Deoxyribonucleic acid methyltransferase 1
DSSP: Define secondary structure of protein
FDR: False discovery rate
ΔGbind: Free energy change of bonding
GI: Gastrointestinal
GROMACS: GROningen machine for chemical simulations
GSCA: Gene set cancer analysis
HCC: Hepatocellular carcinoma
HCV: Hepatitis C virus
HIV: Human immunodeficiency virus
LOO CV: Leave–one–out cross–validation
LD_50_: Median lethal dose
LIHC: Liver hepatocellular carcinoma
MD: Molecular dynamics
MDS: Molecular dynamics simulations
MERS: Middle east respiratory syndrome
MMA: Monomethyl arginine
MMPBSA: Molecular mechanics poisson-boltzmann surface area
P–gp: P-glycoprotein
PAINS: Pan assay interference compounds
PCA: Principal component analysis
PRMT5: Protein arginine methyl transferase 5
PTM: Posttranslational modifications
QTGRACE: Qt-based Grace
RCSB PDB: Research Collaboratory for Structural Bioinformatics Protein Data Bank
Rg: Radius of Gyration
RMSD: Root-mean-square deviation
RMSF: Root–mean–square fluctuation
SASA: Solvent accessible surface area
SDF: Structure-Data File
SDMA: Symmetric dimethylarginine
SN: Staphylococcal nuclease
SND1: Staphylococcal nuclease domain-containing protein 1
SPC: Simple Point Charge
TC: Total clearance
TOP1: [4–(5,6,7,8–Tetrahydro–4 H–cyclohepta[c][1,2]oxazol–3–yl)piperidin–1–yl]–[4–(trifluoromethyl)phenyl]methanone
TOP2: 1–[2–Hydroxy–2–(1–methylsulfonyl–3,4–dihydro–2 H–quinolin–6–yl)ethyl]–4–(4–methylphenyl)piperidin–4–ol
TPSA: Total polar surface area
vdW: van der Waals
VMD: Visual molecular dynamics
