# Supplementary material for: Discovery of novel SND1 inhibitors by in silico–based molecular docking and dynamics simulation methods for managing hepatocellular carcinoma
Source: Sci Rep. 2025 Aug 21;15:30701. doi: 10.1038/s41598-025-14878-0 (PMC12370926; doi:10.1038/s41598-025-14878-0)
Supplement: Supplementary file 1 — Supplementary Material 1 [file 41598_2025_14878_MOESM1_ESM.docx]

**Supplementary Data**

**Table S1.** ADME property and toxicology analysis of TOP1 and TOP2 compounds.

| **Ligand name** | **Ligand name** | **TOP1** | **TOP2** |
| --- | --- | --- | --- |
| Pharmacokinetics | GI absorption | High | High |
|  | BBB permeant | Yes | Yes |
|  | P-gp substrate | Yes | Yes |
|  | CYP1A2 inhibitor | Yes | No |
|  | CYP2C19 inhibitor | Yes | Yes |
|  | CYP2C9 inhibitor | Yes | No |
|  | CYP2D6 inhibitor | Yes | Yes |
|  | CYP3A4 inhibitor | No | No |
| Toxicity | Hepatotoxicity | Inactive | Inactive |
|  | Immunotoxicity | Inactive | Inactive |
|  | Mutagenicity | Inactive | Inactive |
|  | Cytotoxicity | Inactive | Inactive |
| Nuclear receptor signaling pathway | Aryl hydrogen receptor | Inactive | Inactive |
|  | AR | Inactive | Inactive |
|  | ARLBD | Inactive | Inactive |
|  | Aromatase | Inactive | Inactive |
|  | ER-α | Inactive | Inactive |
|  | ERLBD | Inactive | Inactive |
|  | PPAR-γ | Inactive | Inactive |
| Stress response pathway | nrf2/ARE | Inactive | Inactive |
|  | HSEs | Inactive | Inactive |
|  | Mitochondrial membrane potential | Inactive | Inactive |
|  | p53 | Inactive | Inactive |
|  | ATAD5 | Inactive | Inactive |

Abbreviations: ADME = absorption, digestion, metabolism and elimination; AR = Androgen receptor; ARLBD = androgen receptor ligand binding domain; ATAD5 = ATPase family AAA domain-containing protein 5; BBB = blood-brain barrier; CYP = cytochrome P; ERLBD = estrogen receptor ligand binding domain, ER-α = estrogen receptor alpha; GI = gastrointestinal; HSEs = heat shock response elements; nrf2/ARE = nuclear factor erythroid 2-related factor 2/antioxidant regulatory element; P-gp = P-glycoprotein; PPAR-γ = peroxisome proliferator-activated receptor-gamma; p53 = tumor suppressor protein 53; TOP1 = [4-(5,6,7,8-tetrahydro-4H-cyclohepta[c][1,2]oxazol-3-yl)piperidin-1-yl]-[4-(trifluoromethyl)phenyl]methanone; TOP2 = 1-[2-hydroxy-2-(1-methylsulfonyl-3,4-dihydro-2H-quinolin-6-yl)ethyl]-4-(4-methylphenyl)piperidin-4-ol.

**Table S2.** Bioactivity property prediction of TOP1 and TOP2 compounds

| **Name** | **P_a_ ^a^** | **P_i_ ^b^** | **Activity** |
| --- | --- | --- | --- |
| TOP1 | 0.450 | 0.014 | HCV IRES inhibitor |
|  | 0.390 | 0.014 | HIV attachment inhibitor |
|  | 0.306 | 0.240 | Anti-viral (Rhinovirus) inhibitor |
|  | 0.614 | 0.067 | Anti-neurotic |
|  | 0.468 | 0.014 | Analgesic (non-opioid) |
|  | 0.420 | 0.031 | Anti-depressant |
|  | 0.380 | 0.035 | Anti-psychotic |
|  | 0.465 | 0.130 | Anti-ischemic (cerebral) |
|  | 0.305 | 0.080 | Anti-amyloidogenic |
|  | 0.345 | 0.147 | Anti-dyskinetic |
|  | 0.318 | 0.121 | Dementia treatment |
|  | 0.308 | 0.121 | Atherosclerosis treatment |
|  | 0.358 | 0.076 | Menopausal disorder treatment |
|  | 0.412 | 0.031 | Cognition disorder treatment |
|  | 0.750 | 0.005 | Neurodegenerative disease treatment |
|  | 0.758 | 0.004 | Alzheimer’s disease treatment |
| TOP2 | 0.826 | 0.011 | Anti-ischemic (Cerebral) |
|  | 0.344 | 0.042 | Anti-psychotic |
|  | 0.317 | 0.053 | Anti-depressant |
|  | 0.307 | 0.195 | Anti-dyskinetic |
|  | 0.701 | 0.006 | Anti-obesity |
|  | 0.542 | 0.029 | Analgesic |
|  | 0.502 | 0.081 | Acute neurologic disease treatment |
|  | 0.320 | 0.053 | Mood disorder treatment |

Abbreviation: HCV IRES = hepatitis C virus internal ribosome entry site; HIV = human immunodeficiency virus; TOP1 = [4-(5,6,7,8-tetrahydro-4H-cyclohepta[c][1,2]oxazol-3-yl)piperidin-1-yl]-[4-(trifluoromethyl)phenyl]methanone; TOP2 = 1-[2-hydroxy-2-(1-methylsulfonyl-3,4-dihydro-2H-quinolin-6-yl)ethyl]-4-(4-methylphenyl)piperidin-4-ol.

**Table S3.** Hydrogen bond occupancy for the selected compounds that show interaction with SND1.

| **Ligand name** | **Donor** | **Acceptor** | **Occupancy (%)** |
| --- | --- | --- | --- |
| SND1-STD | N823-Side | STD-Side | 2.03 |
|  | Y746-Side | STD-Side | 0.13 |
|  | N768-Side | STD-Side | 0.60 |
|  | Q822-Side | STD-Side | 0.07 |
|  | R819-Side | STD-Side | 0.03 |
|  | V741-Main | STD-Side | 0.07 |
|  | R769-Main | STD-Side | 0.10 |
|  | T688-Side | STD-Side | 0.07 |
|  | N891-Main | STD-Side | 0.07 |
|  | N891-Side | STD-Side | 0.17 |
|  | R732-Main | STD-Side | 0.10 |
|  | R731-Side | STD-Side | 0.33 |
|  | Y728-Side | STD-Side | 0.20 |
|  | K857-Side | STD-Side | 0.13 |
|  | N880-Side | STD-Side | 0.07 |
|  | R749-Side | STD-Side | 0.47 |
|  | R787-Side | STD-Side | 0.07 |
|  | F735-Main | STD-Side | 0.07 |
| SND1-TOP1 | N823-Side | TOP1-Side | 0.63 |
|  | N768-Side | TOP1-Side | 0.07 |
|  | V741-Main | TOP1-Side | 0.03 |
|  | Q699-Side | TOP1-Side | 0.07 |
|  | K683-Side | TOP1-Side | 0.03 |
|  | F686-Main | TOP1-Side | 0.03 |
|  | S681-Main | TOP1-Side | 0.03 |
|  | D846-Main | TOP1-Side | 0.43 |
|  | K848-Side | TOP1-Side | 0.10 |
|  | Q825-Side | TOP1-Side | 0.20 |
| SND1-TOP2 | N823-Side | TOP2-Side | 1.27 |
|  | Q825-Side | TOP2-Side | 0.07 |
|  | Y746-Side | TOP2-Side | 0.30 |
|  | N768-Side | TOP2-Side | 0.37 |
|  | T703-Side | TOP2-Side | 0.50 |
|  | T688-Side | TOP2-Side | 3.0 |
|  | Q706-Side | TOP2-Side | 0.17 |
|  | Y766-Side | TOP2-Side | 1.27 |
|  | R715-Side | TOP2-Side | 0.50 |
|  | Q792-Side | TOP2-Side | 2.47 |
|  | Q699-Side | TOP2-Side | 0.03 |

Abbreviation: SND1 = Staphylococcal nuclease domain-containing protein 1; TOP1 = [4-(5,6,7,8-tetrahydro-4H-cyclohepta[c][1,2]oxazol-3-yl)piperidin-1-yl]-[4-(trifluoromethyl)phenyl]methanone; TOP2 = 1-[2-hydroxy-2-(1-methylsulfonyl-3,4-dihydro-2H-quinolin-6-yl)ethyl]-4-(4-methylphenyl)piperidin-4-ol.

**Table S4.** Summary of the selected compounds showing their interactions that are analyzed through MDS.

| **Name** | **Time (ns)** | **Interaction** | **Distance** | **Category** |  |
| --- | --- | --- | --- | --- | --- |
| SND1-STD | 0 | ASN823:OD1-STD:H3 | 2.561 | Carbon hydrogen bond |  |
|  | 150 | GLU858:OE1-STD:H18 | 1.779 | Conventional hydrogen bond |  |
|  |  | ARG889:O-STD:H12 | 2.865 | Carbon hydrogen bond |  |
|  | 300 | GLU734:OE2-STD:N2 | 4.842 | Attractive charge |  |
|  |  | ARG749:HH12-STD:O1 | 2.080 | Conventional hydrogen bond |  |
|  |  | GLU734:OE2-STD:H14 | 2.078 | Conventional hydrogen bond |  |
| SND1-TOP1 | 0 | ASN768:HD22-TOP1:F23 | 2.349 | Conventional hydrogen Bond;halogen (Fluorine) |  |
|  |  | ASN768:HD22-TOP1:F22 | 2.354 | Conventional hydrogen Bond;halogen (Fluorine) |  |
|  |  | ASN823:HD22-TOP1:O14 | 2.381 | Conventional hydrogen bond |  |
|  |  | ASN768:OD1-TOP1:F22 | 3.122 | Halogen (Fluorine) |  |
|  |  | PHE740-TOP1 | 5.887 | Pi-Pi Stacked |  |
|  |  | TYR766 -TOP1 | 3.975 | Pi-Pi Stacked |  |
|  |  | TYR746-TOP1 | 5.168 | Pi-Pi T-shaped |  |
|  |  | VAL701-TOP1 | 4.914 | Alkyl |  |
|  |  | PHE686-TOP1 | 4.629 | Pi-Alkyl |  |
|  |  | PHE686-TOP1 | 5.4528 | Pi-Alkyl |  |
|  |  | PHE740-TOP1:C6 | 4.404 | Pi-Alkyl |  |
|  |  | TYR746-TOP1:C6 | 4.593 | Pi-Alkyl |  |
|  |  | TYR763-TOP1:C6 | 4.019 | Pi-Alkyl |  |
|  |  | TYR766-TOP1:C6 | 4.179 | Pi-Alkyl |  |
|  | 150 | ASP846:HN-TOP1:O14 | 2.591 | Conventional hydrogen bond |  |
|  |  | TYR682:O-TOP1:F23 | 3.184 | Halogen (Fluorine) |  |
|  |  | LEU827:CD2-TOP1 | 3.508 | Pi-Sigma |  |
|  |  | ALA845-TOP1 | 4.026 | Alkyl |  |
|  |  | LEU827-TOP1:C6 | 4.733 | Alkyl |  |
|  |  | PHE686-TOP1 | 4.761 | Pi-Alkyl |  |
|  |  | PRO684-TOP1 | 5.434 | Pi-Alkyl |  |
|  | 300 | GLN825:OE1-TOP1:H3 | 2.938 | Carbon hydrogen bond |  |
|  |  | CYS826:O-TOP1:H4 | 2.932 | Carbon hydrogen bond |  |
|  |  | CYS826:O-TOP1:H5 | 2.804 | Carbon hydrogen bond |  |
|  |  | VAL701:CG2-TOP1 | 3.739 | Pi-Sigma |  |
|  |  | ALA845-TOP1898 | 4.503 | Alkyl |  |
|  |  | VAL701-TOP1:C6 | 4.892 | Alkyl |  |
| SND1-TOP2 | 0 | PHE740-TOP2 | 5.459 | Pi-Pi stacked |  |
|  |  | TYR766-TOP2 | 4.291 | Pi-Pi stacked |  |
|  |  | PHE686-TOP2 | 5.336 | Pi-Pi T-shaped |  |
|  |  | PHE740-TOP2:C31 | 3.759 | Pi-Alkyl |  |
|  |  | TYR746-TOP2:C31 | 5.074 | Pi-Alkyl |  |
|  |  | TYR763-TOP2:C31 | 4.717 | Pi-Alkyl |  |
|  |  | TYR766-TOP2:C31 | 4.854 | Pi-Alkyl |  |
|  | 150 | MET714:SD-TOP2:H23 | 2.449 | Conventional hydrogen bond |  |
|  |  | GLN699:OE1-TOP2:H5 | 3.014 | Carbon hydrogen bond |  |
|  |  | MET711:CE-TOP2 | 3.983 | Pi-Sigma |  |
|  |  | ILE718:CD-TOP2 | 3.920 | Pi-Sigma |  |
|  |  | ARG715-TOP2:C31 | 4.439 | Alkyl |  |
|  |  | ILE718-TOP2:C31 | 4.753 | Alkyl |  |
|  |  | LEU707-TOP2 | 5.072 | Alkyl |  |
|  |  | MET714-TOP2 | 4.823 | Pi-Alkyl |  |
|  | 300 | ARG715:HH11-TOP2:O24 | 2.307 | Conventional hydrogen bond |  |
|  |  | ARG715:CD-TOP2:O24 | 3.597 | Carbon hydrogen bond |  |
|  |  | GLN706:O-TOP2:H13 | 2.991 | Carbon hydrogen Bond |  |
|  |  | ARG715:NH1-TOP2 | 2.985 | Pi-cation |  |
|  |  | MET711-TOP2 | 4.742 | Alkyl |  |
|  |  | ILE718-TOP2:C31 | 4.349 | Alkyl |  |
|  |  | TRP745-TOP2:C31 | 5.490 | Pi-Alkyl |  |
|  |  | TYR746-TOP2 | 5.465 | Pi-Alkyl |  |

Abbreviation: SND1 = Staphylococcal nuclease domain-containing protein 1, TOP1 = [4-(5,6,7,8-tetrahydro-4H-cyclohepta[c][1,2]oxazol-3-yl)piperidin-1-yl]-[4-(trifluoromethyl)phenyl]methanone; TOP2 = 1-[2-hydroxy-2-(1-methylsulfonyl-3,4-dihydro-2H-quinolin-6-yl)ethyl]-4-(4-methylphenyl)piperidin-4-ol.

**Table S5.** Summary of the selected compounds showing their interactions that are analyzed through MDS

| **S.No** | **PubChem ID** | **Canonical Smiles** | **Vina Score (kcal/mol)** |
| --- | --- | --- | --- |
| TOP1 | 92413470 | C1CCC2=C(ON=C2CC1)C3CCN(CC3)C(=O)C4=CC=C(C=C4)C(F)(F)F | -10.4 |
| TOP2 | 46954798 | CC1=CC=C(C=C1)C2(CCN(CC2)CC(C3=CC4=C(C=C3)N(CCC4)S(=O)(=O)C)O)O | -10.3 |
| TOP3 | 110212857 | CC1=CC=C(C=C1)CC(=O)NC2CC3(CCC(=O)N(CC3)C)OC4=CC=CC=C24 | -10.2 |
| TOP4 | 92558433 | CC1=CC=C(C=C1)C2=CC=CC=C2C[C@@H]3CN(CCNC3=O)C(=O)C4=CC=CC=C4 | -10.2 |
| TOP5 | 16671632 | C1CCC(CC1)NC(=O)[C@H]2C=CCN2C(=O)C3=CC=CC=C3F | -10.1 |
| TOP6 | 16669238 | C1CN(CCC12NC(=O)C3=CC=CC=C3O2)C(=O)CN4C(=O)C5=CC=CC=C5C=N4 | -9.9 |
| TOP7 | 95803799 | CC1=CC=C(C=C1)C2(CCCC2)C(=O)N3CCC(CC3)N4C5=C(C=CC=N5)OC4=O | -9.9 |
| TOP8 | 110220686 | CN1C(=O)C=CC(=N1)C(=O)N2CC3CCCC(C3C2)(C4=CC(=CC=C4)C(F)(F)F)O | -9.8 |
| TOP9 | 46954920 | C1COC2=C(C=C(C=C2)C(CN3CCC(CC3)(C4=CC=C(C=C4)C(F)(F)F)O)O)OC1 | -9.8 |
| TOP10 | 92558071 | CC1=CC=C(C=C1)S(=O)(=O)N2CC[C@H]3[C@@H](C2)[C@@H](CN3C(=O)C)C4=CC=CC(=C4)C | -9.7 |
| TOP11 | 92418724 | CC1=CC=C(C=C1)CC(=O)N2CCCC[C@@H]2C3=CC4=NC(=C(C(=O)N4N3)C)C | -9.6 |
| TOP12 | 110218978 | C1CN(CCC12C(C(=O)NC2=O)C3=CC=C(C=C3)F)C(=O)C4=CC=CC=C4F | -9.6 |
| TOP13 | 92419523 | CC1=NN(C(=O)C2=CC=CC=C12)CC(=O)N3[C@@H]4CC[C@H]3CC(C4)C5=CC=C(C=C5)F | -9.6 |
| TOP14 | 92555944 | CC1=CC=C(C(=O)N1)C(=O)N2CCC[C@H](C2)C3=NC4=C(C(=NO4)C)C(=C3)C(F)(F)F | -9.6 |
| TOP15 | 124350199 | CC1=CC2=C(C=C1)C(=C(N2)C)[C@H](CN3CCC[C@](CC3)(C4=CC=C(C=C4)F)O)O | -9.5 |
| TOP16 | NA | O=C1N[C@]5(Nc2ncccc12)[C@@]6([H])CN(CC(=O)N3C[C@@]([H])(CCC3)c4ccccc4)C(=O)C[C@@]56[H] | -9.5 |
| TOP17 | 92406880 | C1=CC=C(C=C1)C2(CC2)C(=O)N3CCC[C@@H]3CN4C5=CC=CC=C5OC4=O | -9.5 |
| TOP18 | 92574097 | C1CN(C[C@@H]2[C@H]1N(C[C@H]2C3=CC=C(C=C3)F)C(=O)C4=CC=CC=C4)C(=O)C5=CC=CC=N5 | -9.5 |
| TOP19 | 110213281 | CC1CN(CC(O1)C)C(=O)CN2CC3(CCN(CC3)C)OC4=C(C2=O)C=C(C=C4)C | -9.5 |
| TOP20 | 92408839 | CC1=CC=C(C=C1)S(=O)(=O)N2CCC[C@]3(C2)CN(CC4=C(O3)C=CC(=C4)C)C(=O)C | -9.5 |
| TOP21 | 92558305 | CC1=CC=C(C=C1)C2=CC=CC(=C2)C[C@@]3(CCN(C3)C(=O)C4=CN=CC=C4)C(=O)NC(C)C | -9.5 |
| TOP22 | NA | O=[S]1(=O)NC3(Nc2ccccc12)CCN(CC3)C(=O)C4(CC4)c5ccc(C)cc5 | -9.5 |
| TOP23 | 95797075 | CN(C)C(=O)[C@H]1CN(C[C@@]12CCC3=CC=CC=C3C(=O)N2)CC4=C(C(=CC=C4)F)F | -9.5 |
| TOP24 | 92408559 | CC1=CC2=C(C=C1)OC3(CCOCC3)CN(C2=O)CC(=O)N4CCC(CC4)O | -9.5 |
| TOP25 | 92556399 | CC1=CC=C(C=C1)C(=O)N2CCC[C@H](C2)C3=NC(=NC=C3C4=CN=CC=C4)N5CCOCC5 | -9.5 |
| TOP26 | 92559731 | CC1=CC2=C(C=C1)OC3(CCN(CC3)C)CN(C2=O)CC(=O)N4CCC(CC4)O | -9.5 |
| TOP27 | 95793471 | C[C@@]12CC[C@@H](C[C@@H]1C(=O)NCC3=CC=CC=C3F)[C@@]4(C2)NC5=CC=CC=C5C(=O)N4 | -9.5 |
| TOP28 | 136681175 | CC1=CC2=NC(=C(C=C2C=C1)CN3CCC4=C(C3)N=C(NC4=O)C5=CN=CC=C5)OC | -9.5 |
| TOP29 | 92559740 | CC1=CC2=C(C=C1)OC3(CCN(CC3)C)CN(C2=O)CC(=O)N4CCCCC4 | -9.5 |
| TOP30 | 110213310 | CC1=CC2=C(C=C1)OC3(CCN(CC3)C)CN(C2=O)C(C)C(=O)NC4CCCOC4 | -9.4 |
| TOP31 | 95797063 | CN(C)C(=O)[C@H]1CN(C[C@@]12CCC3=CC=CC=C3C(=O)N2)CC4=CC(=CC=C4)F | -9.4 |
| TOP32 | 95792443 | CC1=CC=C(C=C1)C2(CCCC2)C(=O)N[C@@H](C)CN3C(=O)C=C4CCCCC4=N3 | -9.4 |
| TOP33 | 92562237 | CC1=CC=CC=C1C2=NOC(=N2)[C@H]3CN(CCO3)C(=O)C4=CC5=C(C=C4)N(C(=C5C)C)C | -9.4 |
| TOP34 | 155900978 | CC1=CC=C(C=C1)CNC(=O)CN2CCC3(CCC2=O)NC(=O)C4=CC=CC=C4O3 | -9.4 |
| TOP35 | 92406896 | CC1=CC=C(C=C1)C2(CCCC2)C(=O)N[C@H](C)CN3C(=O)C=C4CCCCCC4=N3 | -9.4 |
| TOP36 | 136681198 | C1CN(CC2=C1C(=O)NC(=N2)C3=CC=NC=C3)CC4=CC=C(C=C4)C(F)(F)F | -9.4 |
| TOP37 | 110213373 | CC1=CC2=C(C=C1)OC3(CCOCC3)CN(C2=O)C(C)C(=O)NCC4=CC=CC=N4 | -9.4 |
| TOP38 | 95849899 | CC1=CC=C(C=C1)CC(=O)N2CCC[C@@H](C2)C3=NN=C(O3)C4=NC=CC5=CC=CC=C54 | -9.4 |
| TOP39 | 92559721 | C[C@@H]1CCC[C@H](N1C(=O)CN2CC3(CCN(CC3)C)OC4=C(C2=O)C=C(C=C4)C)C | -9.4 |
| TOP40 | 16673533 | CC1=CC2=C(C=C1C)OC3(CCN(CC3)CC(C4=CN(C5=CC=CC=C54)C)O)CC2O | -9.4 |
| TOP41 | 51501838 | CC1=CC2=C(C=C1)OC3(CCN(CC3)C[C@H](C4=C(NC5=CC=CC=C54)C)O)C[C@@H]2O | -9.4 |
| TOP42 | 110213291 | CC1=CC2=C(C=C1)OC3(CCN(CC3)C)CN(C2=O)C(C)C(=O)NC4CCCC4 | -9.4 |
| TOP43 | 17358828 | CC(C)CNC(=O)CCC1=CC=C(C=C1)NC(=O)CC(C2=CC=CC=C2)C3=CC=CC=C3 | -9.4 |
| TOP44 | 92558299 | CC1=CC=C(C=C1)C2=CC=CC(=C2)C[C@@]3(CCN(C3)C(=O)C4=CC=NN4)C(=O)N(C)C | -9.4 |
| TOP45 | 92406919 | CC1=CC(=CC(=C1)C(=O)N[C@H](C)CN2C(=O)C=C3CCCCCC3=N2)C | -9.4 |
| TOP46 | 92592355 | CCNC(=O)[C@]1(CCN(C1)C(=O)C2=C(N=CS2)C)CC3=CC(=CC=C3)C4=CC=C(C=C4)C | -9.4 |
| TOP47 | 92407816 | C1CN(CCC12NC3=CC=CC=C3C4=NCCN24)CC5=NNN=C5C6=CC=CC=C6 | -9.4 |
| TOP48 | 136681128 | C1CN(CC2=C1C(=O)NC(=N2)C3=CN=CC=C3)CC4=CC=C(C=C4)C(F)(F)F | -9.4 |
| TOP49 | 46953634 | CC1=NN2C(=CC(=NC2=C1)C3CCCN(C3)C(=O)C4=CC5=C(CCC5)C=C4)C(F)(F)F | -9.4 |
| TOP50 | 110215011 | CC1=CC2=C(C=C1)OC=C(C2=O)CN3C4CCC3C5=C(C4)N6C(=CC(=N6)C)N=C5 | -9.4 |
| TOP51 | 92591722 | CCN1CCN(CC1)C(=O)CN2CC3(CCN(CC3)C)OC4=C(C2=O)C=C(C=C4)C | -9.4 |
| TOP52 | 110213294 | CC1CCCN(C1)C(=O)C(C)N2CC3(CCN(CC3)C)OC4=C(C2=O)C=C(C=C4)C | -9.4 |
| TOP53 | 92559745 | CC1=CC2=C(C=C1)OC3(CCN(CC3)C)CN(C2=O)CC(=O)N4CCN(CC4)C | -9.3 |
| TOP54 | 95848768 | C[C@H](CN1CCN(C(=O)[C@@H](C1)CC2=CC=CC=C2C3=CC=NC=C3)C)C4=CC=CC=C4 | -9.3 |
| TOP55 | 16675054 | CC(C(=O)NC1CC2(CCCC2)OC3=CC=CC=C13)N4C(=O)C5=CC=CC=C5C=N4 | -9.3 |
| TOP56 | 95849538 | CC1=CC=C(C=C1)C2=NO[C@@H](C2)CC3(CCOCC3)C(=O)NC4=C(C=C(C=C4)C)C | -9.3 |
| TOP57 | 92568306 | C1C[C@@H]2C(=O)N(C3=C(N2C1)N=CC=C3)CC(=O)NCC4=CC(=CC=C4)C(F)(F)F | -9.3 |
| TOP58 | 92608553 | CN1CCN(C[C@H](C1=O)CC2=CC=CC=C2C3=CC=CC=C3OC)C(=O)C4=CC(=CC=C4)F | -9.3 |
| TOP59 | 16669553 | CC1=CC=C(C=C1)C(C)C(=O)N2CCC(CC2)C3=CC=NC4=NC(=NN34)C(F)(F)F | -9.3 |
| TOP60 | 95792432 | C[C@@H](CN1C(=O)C=C2CCCCC2=N1)NC(=O)C3=CC=C(C=C3)C(F)(F)F | -9.3 |
| TOP61 | 92591565 | CCN1CCN(C[C@H](C1=O)CC2=CC=CC=C2C3=CC=C(C=C3)C)C(=O)C4=C(NN=C4)C | -9.3 |
| TOP62 | 95797287 | CC1=CC=C(C=C1)C2=NN(C(=O)C3=CC4=CC=CC=C4N3C2)CC(=O)N(C)C | -9.3 |
| TOP63 | 46956359 | CC1=CC=C(C=C1)[C@@H]2CN(C[C@H]2C3=CSN=N3)CC(=O)N4CCCN(CC4)C(=O)C | -9.3 |
| TOP64 | 92547971 | C#CCNC(=O)[C@@]1(CCN(C1)CC2=CC=CC=C2)CC3=CC=CC=C3C4=CN=CC=C4 | -9.3 |
| TOP65 | 92559732 | CC1=CC2=C(C=C1)OC3(CCN(CC3)C)CN(C2=O)CC(=O)N4CCCC(C4)(C)C | -9.3 |
| TOP66 | 135719803 | CCC1=C2C(=CC=C1)C(=CN2)CN3CCC4=C(C3)N=C(NC4=O)N5CCOCC5 | -9.3 |
| TOP67 | 136763841 | CC1=NC(=CC(=O)N1)[C@@H]2CN(C[C@H]2C3=CC=CC=C3F)C(=O)CC4=CC=C(C=C4)F | -9.3 |
| TOP68 | 92572718 | C1CO[C@H](CN1C(=O)CCC2=CC=CC=C2F)C3=NC(=NO3)C4=CC(=CC=C4)C(F)(F)F | -9.3 |
| TOP69 | 46955464 | CC1=CC=C(C=C1)C2=NOC3C2CN(C3)C(=O)C4CCS(=O)(=O)C4 | -9.3 |
| TOP70 | 110213776 | CC1=CC2=C(C=C1)OC3(CCC4C3C4C(=O)N5CCC(CC5)C(=O)N)CC2=O | -9.3 |
| TOP71 | 92559808 | CC1=CC2=C(C=C1)OC3(CCOCC3)CN(C2=O)CC(=O)NCC4=CC=CC=N4 | -9.3 |
| TOP72 | 92558072 | CC1=CC=C(C=C1)S(=O)(=O)N2CC[C@H]3[C@@H](C2)[C@@H](CN3C(=O)C)C4=CC(=CC=C4)OC | -9.3 |
| TOP73 | 92408564 | CC1=CC2=C(C=C1)OC3(CCOCC3)CN(C2=O)CC(=O)NC4=CC(=NN4C(C)C)C | -9.3 |
| TOP74 | 92591723 | CCN1CCN(CC1)C(=O)CN2CC3(CCOCC3)OC4=C(C2=O)C=C(C=C4)C | -9.3 |
| TOP75 |  |  | -9.3 |
| TOP76 | 92572693 | C1CN(C[C@H](C(=O)N1)CC2=CC=CC=C2C3=CC=NC=C3)C(=O)C4=CC(=CC=C4)F | -9.3 |
| TOP77 | 110213584 | CC1=CC2=C(C=C1)OC3(CCCN(C3)C(=O)CC4=CC=C(C=C4)F)CN(C2)C | -9.3 |
| TOP78 | 92574099 | C1CN(C[C@@H]2[C@H]1N(C[C@H]2C3=CC=C(C=C3)F)C(=O)C4=CC=CC=C4)C(=O)C5=CN=CC=C5 | -9.3 |
| TOP79 | 92416069 | CC1=CC2=C(C=C1)OC3(CCN(CC3)C)CN(C2=O)CC(=O)N4CCNC(=O)C4 | -9.3 |
| TOP80 | 46954714 | CC1CC2=C(N1S(=O)(=O)C)C=CC(=C2)C(=O)CN3CCC(CC3)(C4=CC=CC=C4F)O | -9.3 |
| TOP81 | 92558152 | CC1=CC=C(C=C1)S(=O)(=O)N[C@H](C)C(=O)N2CC3=CC=CC=C3OC4(C2)CCOCC4 | -9.2 |
| TOP82 | 46953162 | COC1=CC2=C(CN(CC2)C(=O)C3CC4CCC3C[C@@]45NC6=CC=CC=C6C(=O)N5)C=C1 | -9.2 |
| TOP83 | 95797652 | CC1=CC=C(C=C1)[C@H](C)C(=O)N2CCC[C@H](C2)C3=C(C=NC4=NC=NN34)S(=O)(=O)C | -9.2 |
| TOP84 | NA | O=C1N[C@]3(Nc2ccccc12)C[C@@]4(C)[C@]5(C[C@]3([H])C[C@@]45[H])C(=O)NCc6ccccc6[F] | -9.2 |
| TOP85 | 2587187 | CC1=CC(=NC2=NC(=NN12)SCC(=O)N(CCC#N)C3=CC=CC=C3)C | -9.2 |
| TOP86 | 92598466 | CC1=C(SC=N1)C(=O)N2CC[C@@H]3[C@H](C2)[C@H](CN3C(=O)C4=CC=CC=C4)C5=CC=C(C=C5)F | -9.2 |
| TOP87 | 16669444 | CC1=NOC(=C1)C2=CN=CN=C2C3CCN(CC3)C(=O)C4(CCCC4)C5=CC=CC=C5F | -9.2 |
| TOP88 | NA | O=C(/N=C(\c1ccc(C)cc1)c2ccncc2)Cc4cc3C[C@@](C)([H])Oc3cc4 | -9.2 |
| TOP89 | 136777688 | CC1=CC=C(C=C1)C(C)C(=O)N2CCC[C@@H]2CN3C4=C(C(=O)NC(=N4)C)N=N3 | -9.2 |
| TOP90 | 92556590 | CC1=CC=C(C=C1)[C@H](C)C(=O)N2CCC[C@]3(C2)CNC(=O)C4=CC=CC=C4O3 | -9.2 |
| TOP91 | 92599548 | COC1=CC=CC(=C1)C(=O)N2CCNC(=O)[C@@H](C2)CC3=CC=CC=C3C4=CC=NC=C4 | -9.2 |
| TOP92 | 92556467 | CC1=CC=C(C=C1)C(=O)N2CCO[C@@H](C2)C3=NC(=NO3)C4=NC=CC=N4 | -9.2 |
| TOP93 | 92574101 | C1CN(C[C@@H]2[C@H]1N(C[C@H]2C3=CC=C(C=C3)F)C(=O)C4=CC=CC=C4)C(=O)C5=CC=NC=C5 | -9.2 |
| TOP94 | 92608546 | CN1CCN(C[C@H](C1=O)CC2=CC=CC=C2C3=CC=CC=C3)C(=O)C4=CC=CC=C4OC | -9.2 |
| TOP95 | 46954298 | CC1=NOC(=C1)C2=CC(=O)NN=C2C3CCN(CC3)C(=O)C4=CC=C(C=C4)C(F)(F)F | -9.2 |
| TOP96 | 46954298 | CC1=NOC(=C1)C2=CC(=O)NN=C2C3CCN(CC3)C(=O)C4=CC=C(C=C4)C(F)(F)F | -9.2 |
| TOP97 | 92591555 | CCN1CCN(C[C@H](C1=O)CC2=CC=CC=C2C3=CC=C(C=C3)C)C(=O)CCN4C=NN=N4 | -9.2 |
| TOP98 | 92558870 | CC1=NC=C(C=C1)C(=O)N2CC[C@H]3[C@@H](C2)[C@@H](CN3C(=O)C4=CC=CC=N4)C5=CC=C(C=C5)F | -9.2 |
| TOP99 | 92560474 | CC1=CC(=CC=C1)C(=O)N2CCCC[C@H]2CCN3C(=O)C=C4CCCCCC4=N3 | -9.2 |
| TOP100 | NA | O=C1N5C(=NN1CCNC(=O)[C@@]3([H])[C@@]2([H])C(=O)N(C)C[C@@]24O[C@]3([H])CC4)C(C)=Cc6ccc(C)cc56 | -9.2 |

**Supplementary Figures**

**
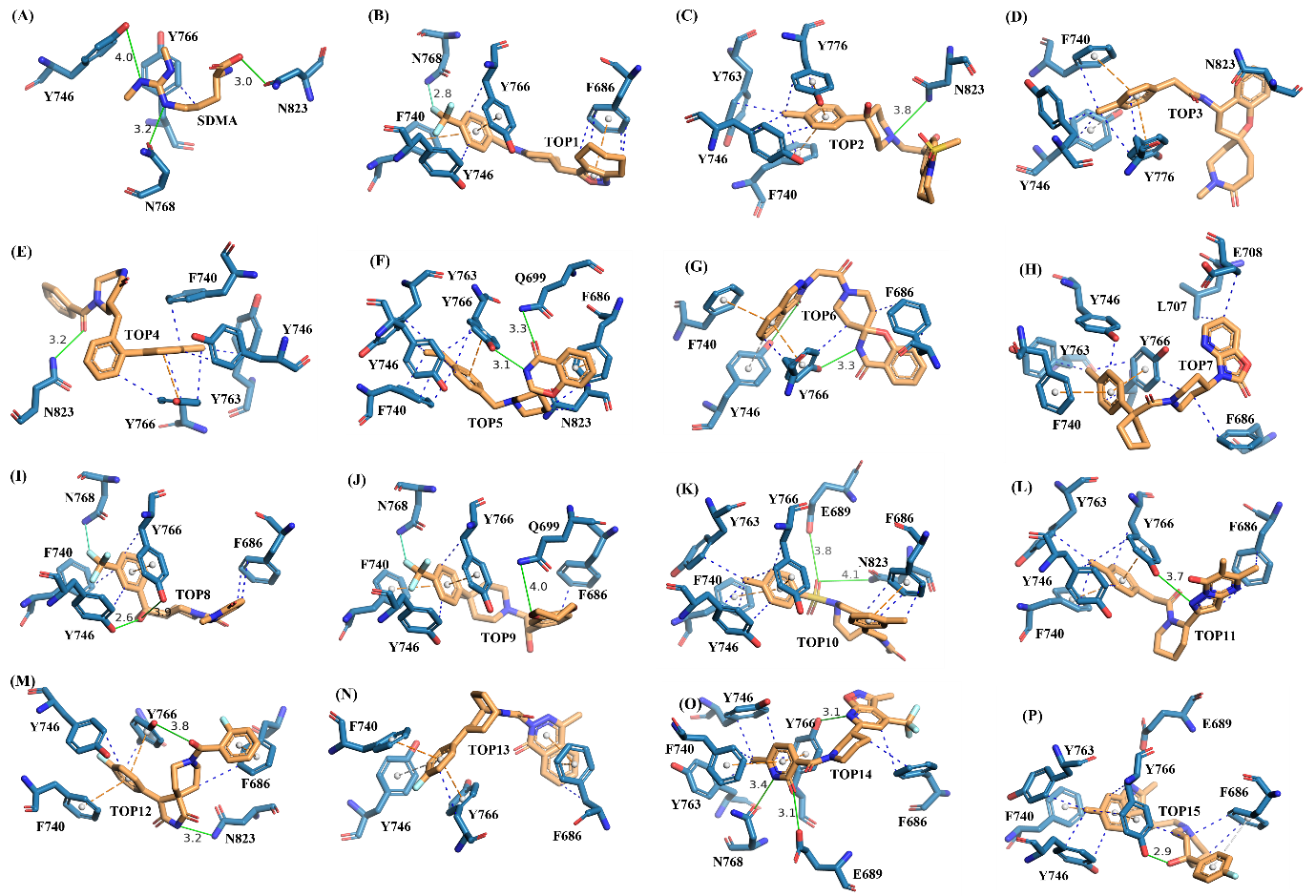
**

**Figure S1**. 3D-stick representation of the active site residues interacting with each ligand. (A) STD, (B-D) TOP1 to TOP3. (E-H) TOP4 to TOP7. (I-L) TOP8 to TOP11. (M-P) TOP12 to TOP15. For interpretation, the blue sticks indicate the active site residues, and the orange sticks indicate the ligand. The green color line represents the hydrogen bond. A blue color line represents the hydrophobic interactions. The orange line represents the cation-pi interaction, and the grey line represents the pi-pi interaction. Abbreviation: TOP1 = [4-(5,6,7,8-tetrahydro-4H-cyclohepta[c][1,2]oxazol-3-yl)piperidin-1-yl]-[4-(trifluoromethyl)phenyl]methanone; TOP2 = 1-[2-hydroxy-2-(1-methylsulfonyl-3,4-dihydro-2H-quinolin-6-yl)ethyl]-4-(4-methylphenyl)piperidin-4-ol.


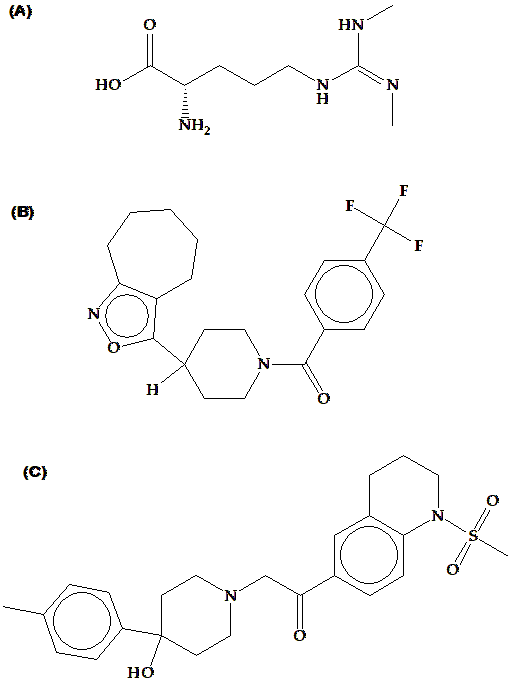


**Figure S2.** 2D structural representation of the selected compounds that are used in this study. (A) STD (Symmetrically demethylated arginine), (B) TOP1 ([4-(5,6,7,8-tetrahydro-4H-cyclohepta[c][1,2]oxazol-3-yl)piperidin-1-yl]-[4-(trifluoromethyl)phenyl]methanone), and (C) TOP2 (1-[2-hydroxy-2-(1-methylsulfonyl-3,4-dihydro-2H-quinolin-6-yl)ethyl]-4-(4-methylphenyl)piperidin-4-ol).


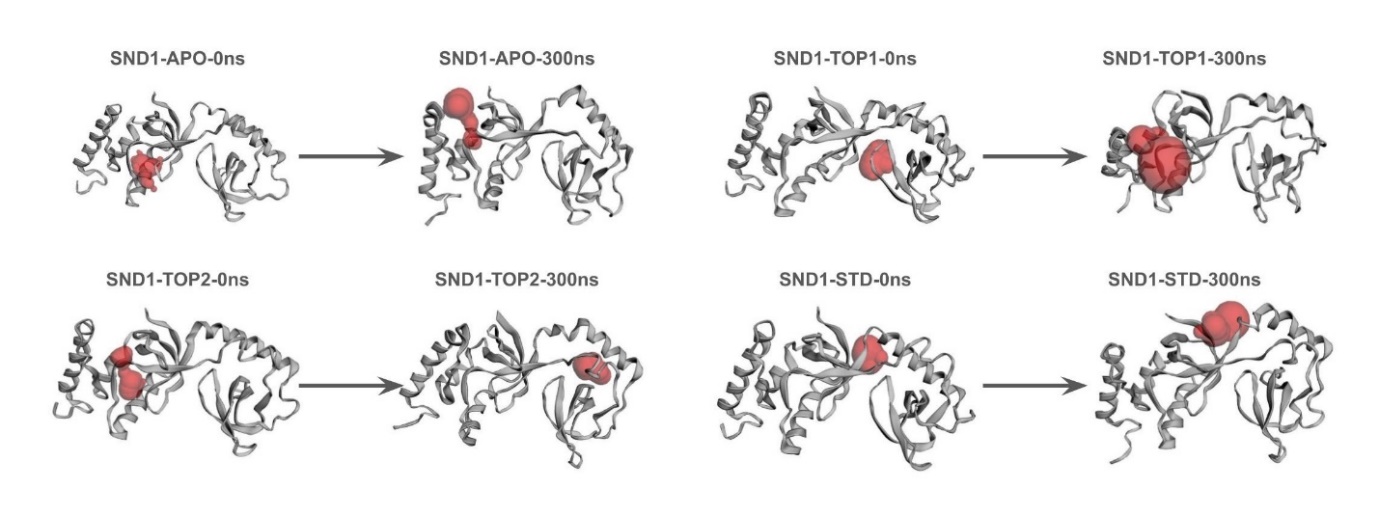


**Figure S3.** Structural representation showing the cavity formed in apo and each ligand-protein bound complex. Abbreviation: SND1 = Staphylococcal nuclease domain-containing protein 1; TOP1 = [4-(5,6,7,8-tetrahydro-4H-cyclohepta[c][1,2]oxazol-3-yl)piperidin-1-yl]-[4-(trifluoromethyl)phenyl]methanone; TOP2 = 1-[2-hydroxy-2-(1-methylsulfonyl-3,4-dihydro-2H-quinolin-6-yl)ethyl]-4-(4-methylphenyl)piperidin-4-ol.

**
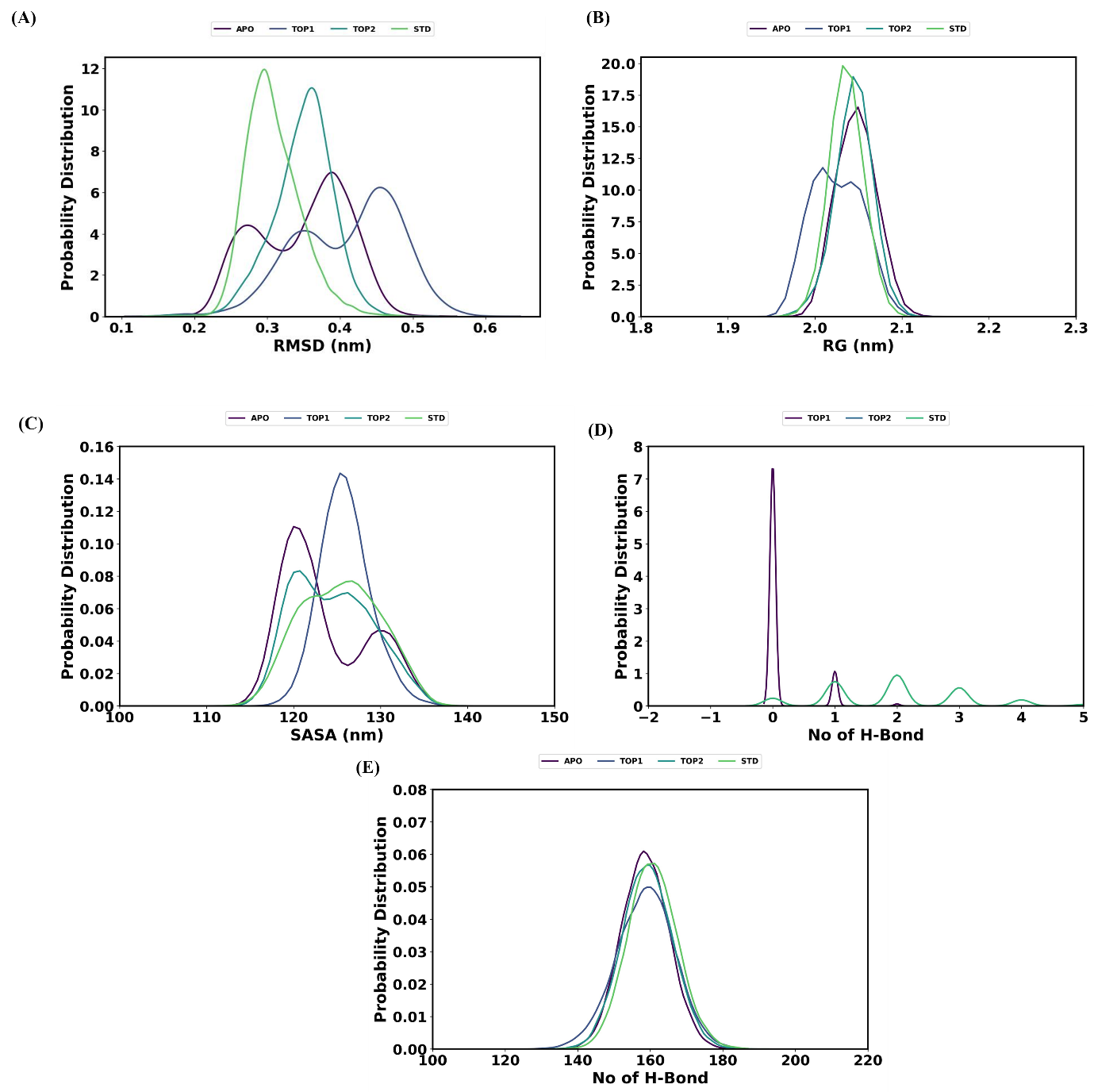
Figure S4.** Probability distribution plot of STD, TOP1, and TOP2-docked complexes attained during MDS analysis. (A) showing the probability distribution of RMSD of APO, STD, TOP1 and TOP2, (B) depicts the probability distribution of Rg of APO, STD, TOP1, and TOP2, (C) demonstrates the probability distribution of SASA of APO, STD, TOP1 and TOP2, (D) indicates the probability distribution of H-bonds (Inter) formed in STD, TOP1 and TOP2-complexes and (E) represents the Intra (overall) probability distribution of H-bonds formed in APO, STD, TOP1, and TOP2 systems. Abbreviation: MDS = molecular dynamics simulations; RMSD = root-mean-square deviation; Rg = Radius of Gyration; SASA = solvent accessible surface area.


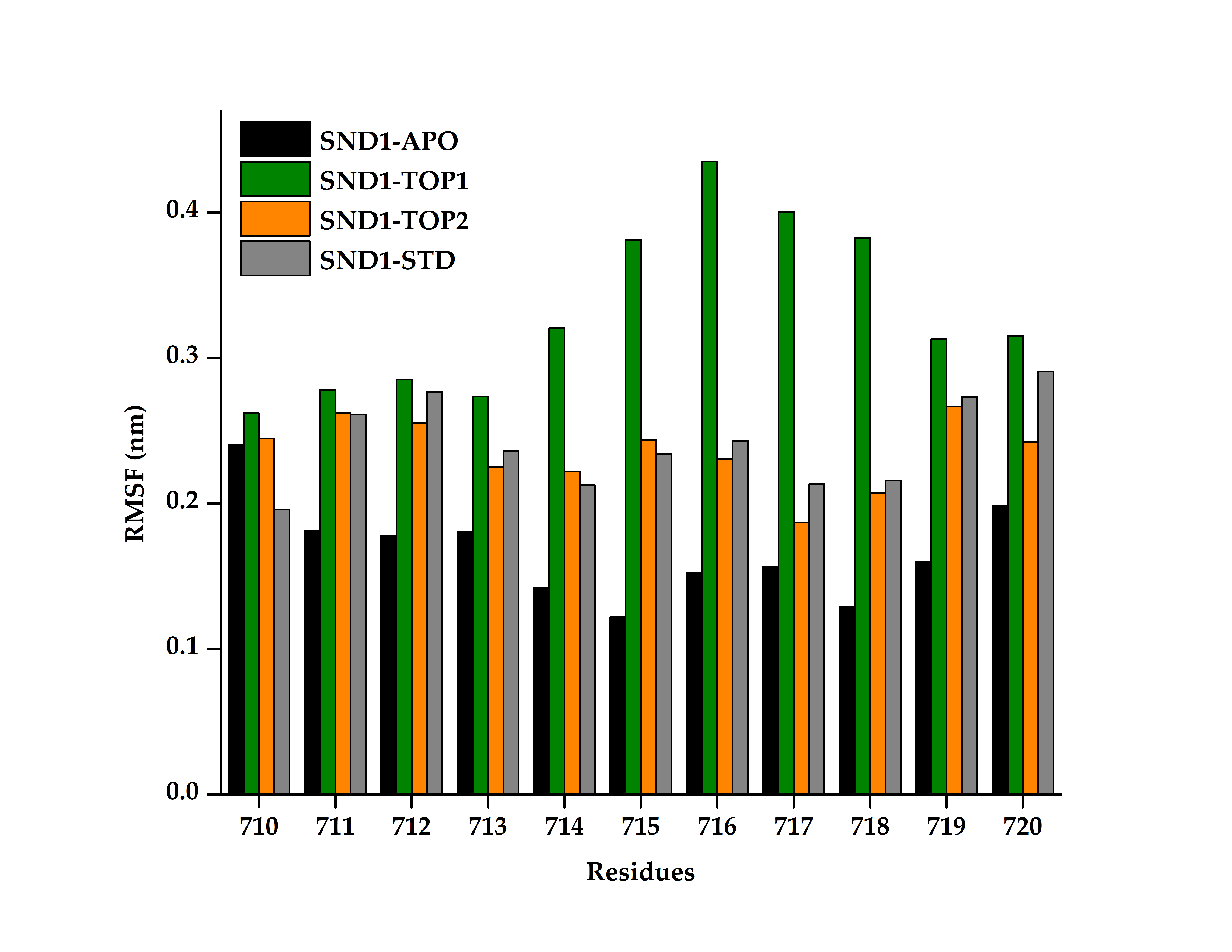
**Figure S5.** RMSF analysis of the specific residues shows various APO and ligand-bound protein complexes fluctuations. Abbreviation: RMSF = root-mean-square fluctuation; SND1 = Staphylococcal nuclease domain-containing protein 1; TOP1 = [4-(5,6,7,8-tetrahydro-4H-cyclohepta[c][1,2]oxazol-3-yl)piperidin-1-yl]-[4-(trifluoromethyl)phenyl]methanone; TOP2 = 1-[2-hydroxy-2-(1-methylsulfonyl-3,4-dihydro-2H-quinolin-6-yl)ethyl]-4-(4-methylphenyl)piperidin-4-ol.
